# Chemical Constituents from *Euphorbia esula*

**DOI:** 10.3390/plants14182822

**Published:** 2025-09-09

**Authors:** Defeng Yan, Miaomiao Zhang, Yuqing Song, Liu Liu, Nurmirza Begmatov, Orzimat Turdimatovich Turginov, Bo Zhao, Hequn Yang, Guoan Zou

**Affiliations:** 1 State Key Laboratory Basis of Xinjiang Indigenous Medicinal Plants Resource Utilization, Xinjiang Technical Institute of Physics and Chemistry, Chinese Academy of Sciences, Urumqi 830011, China; yandefeng21@mails.ucas.ac.cn (D.Y.); zhangmiaomiao20@mails.ucas.ac.cn (M.Z.); songyuqing22@mails.ucas.ac.cn (Y.S.); liuliu@ms.xjb.ac.cn (L.L.); zhaobo@ms.xjb.ac.cn (B.Z.); yanghq@ms.xjb.ac.cn (H.Y.); 2 University of Chinese Academy of Sciences, Beijing 100049, China; 3 S. Yu. Yunusov Institute of the Chemistry of Plant Substances, Academy of Sciences of the Republic of Uzbekistan, 77, M. Ulugbek Str., Tashkent 100170, Uzbekistan; nurmirza87@yahoo.com; 4 Institute of Botany, Academy of Sciences of the Republic of Uzbekistan, Tashkent 100047, Uzbekistan; orzimat@mail.ru

**Keywords:** *Euphorbia esula*, Euphorbiaceae, alkaloid, norisoprenoid, ECD calculation, anti-inflammatory activity

## Abstract

*Euphorbia esula* is widely distributed across China, Central Asia and other regions worldwide. For centuries, it has been applied in folk and traditional medicine as a cure for diverse ailments. Nevertheless, the bioactive components responsible for anti-inflammatory and cytotoxic effects remain incompletely identified. In this study, two undescribed chemical constituents, a pyrrole alkaloid (**1**) and a loliolide analogue (**2**), alongside nine known components (**3**–**11**) were separated from the aerial parts of *Euphorbia esula* indigenous to Uzbekistan. Their chemical structures were comprehensively elucidated utilizing HRESIMS, NMR, IR and UV spectroscopy. Corresponding absolute configurations were determined based on comparison of experimental and calculated ECD data. Compounds **3**–**11** were firstly isolated from *Euphorbia esula*, among which **4**, **5**, **7** and **9**–**11** were yielded from the genus *Euphorbia* for the first time. Chemically, the discovery of various skeletons covering pyrrole alkaloids (**1**, **9**), norisoprenoids (**2**–**8**), furanone (**10**) and unusual cyclooct-2-enone (**11**) particularly highlighted the structural diversity. Bioactivity assays revealed that some compounds (**1**, **3**, **5**, **6**, **7** and **8**) exhibited certain anti-inflammatory effects via inhibiting the NO release in LPS-induced RAW 264.7 macrophages.

## 1. Introduction

Among the most taxonomically diverse families within the higher plants, the Euphorbiaceae encompasses approximately 300 genera and 8000 species, demonstrating tremendous species richness across a wide range of ecological habitats [1]. *Euphorbia* L. ranks among the most species-rich genera in the angiosperm clade, along with *Astragalus*, *Bulbophyllum*, *Psychotria*, *Carex* and *Begonia*, comprising approximately 2160 recognized species further subdivided into multiple subgenera and taxonomic sections. As a typical characteristic of this genus, the production of a milky, irritant latex serves as a key diagnostic feature in taxonomic classification. *Euphorbia* species display a worldwide distribution across both tropical and temperate mainland ecosystems, with remarkable morphological plasticity from tiny annual or perennial herbal forms to lianas, large desert succulents, woody shrubs or even arborescent species [2,3].

As a perennial herb belonging to the genus *Euphorbia*, *Euphorbia esula* L. represents a distinct growth habit, attaining a characteristic stature during its ontogenetic development [4,5]. Morphologically, it is distinguished by its green foliar structure and the production of a milky white latex upon tissue disruption. Geographically, this species demonstrates a wide ecological distribution in various regions of China, Central Asia and Europe, with additional occurrences recorded in other parts of the globe [6]. In previous phytochemical and biological investigations on *E. esula*, a number of chemical constitutions have been brought to light, especially various diterpenoids, including types of jatrophane [7,8], ingenane [9] and lathyrane [10], in addition to triterpenoids [9] and flavonoids [11]. Moreover, the species also produces alkanes, sterols, long-chain alcohols, alkaloids, organic acids and amino acids [12]. These secondary metabolites display a wide range of biological functions involving anti-inflammatory, antimicrobial, anti-osteoclastogenic, anti-tumor, cocarcinogenic and MDR (multidrug resistance) reversal properties [7,8,9].

In the process of our continuous efforts to explore bioactive substances from potential medicinal plants native to Central Asia, the ethanol extract of the aerial parts of *E. esula* was processed applying multiple extraction and isolation methods containing column chromatography in combination with semi-PHPLC. The chemical structural formulas of undescribed compounds (**1**–**2**) and known ones (**3**–**11**) were determined by means of extensive HRESIMS, NMR spectroscopy (HSQC, HMBC, ^1^H–^1^H COSY and NOESY), IR, UV and ECD techniques. Additionally, preliminary evaluations of their anti-inflammatory and cytotoxic activities were also conducted, laying an experimental foundation for the chemical research and medicinal development of this plant species.

## 2. Results

### 2.1. Separation and Structural Characterization for Compounds ***1**–**11***

**Figure 1:**
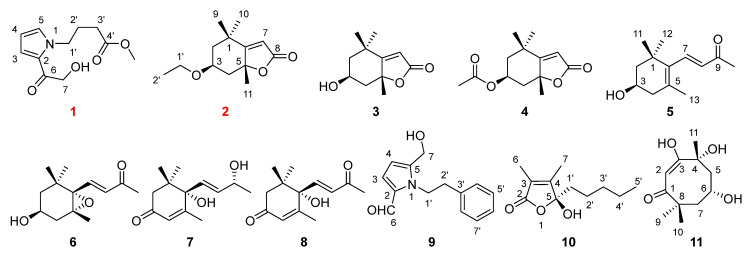
Chemical structures of compounds **1**–**11**.

The 80% ethanol extract from the overground organs of *Euphorbia esula* was carried out via a systematic chromatographic separation over silica gel column chromatography (CC) combined with semi-PHPLC, leading to the isolation of 11 chemical substances (Figure 1). The structural identification of unprecedented components (**1**–**2**) was achieved through the comprehensive elucidation of NMR spectral data in addition to complementary analysis of HRESIMS, IR, UV and ECD spectra. The known constituents (**3**–**11**) were structurally confirmed by contrasting their physicochemical properties and spectroscopic evidence with the reported ones. ^1^H and ^13^C NMR data for undescribed compounds (**1**–**2**) are listed in Table 1.

**Figure 2:**
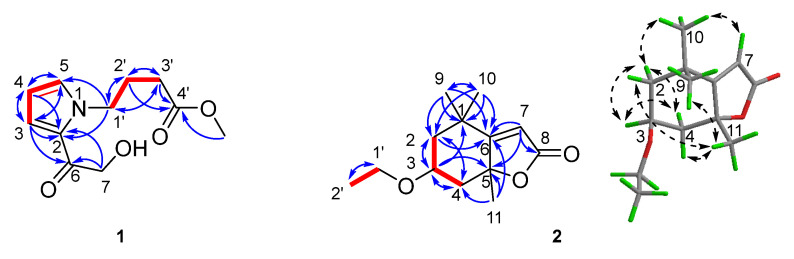
^1^H–^1^H COSY (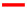), HMBC (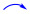) and NOESY (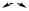) correlations of compounds **1**–**2**.

Methyl 4-[2-(2-hydroxyacetyl)-1*H*-pyrrol-1-yl]butanoate (**1**) was obtained as colourless oil. Its (+)-HRESIMS showed an ion peak of *m*/*z* 226.1070 [M+H]^+^ (calcd for C_11_H_16_NO_4_, 226.1074), indicating the molecular formula C_11_H_15_NO_4_ with five degrees of unsaturation. The ^1^H NMR, ^13^C NMR (Table 1), HSQC and HMBC signals of compound **1** (Figure 1) indicated one ketone carbonyl at *δ*_C_ 189.9 (C-6) and one ester carbonyl at *δ*_C_ 175.1 (C-4′); four olefinic carbons (three protonated) at *δ*_C_ 132.5 (C-5), *δ*_H_ 7.08 (1H, overlapped, H-5); *δ*_C_ 128.1 (C-2), *δ*_C_ 120.5 (C-3), *δ*_H_ 7.07 (1H, overlapped, H-3), *δ*_C_ 109.7 (C-4) and *δ*_H_ 6.17 (1H, dd, *J* = 3.9, 2.7 Hz, H-4); four methylene units (one oxygenated) at *δ*_C_ 65.4 (C-7), *δ*_H_ 4.63 (2H, s, H-7), *δ*_C_ 49.5 (C-1′), *δ*_H_ 4.41 (2H, t, *J* = 6.9 Hz, H-1′), *δ*_C_ 31.5 (C-3′), *δ*_H_ 2.28 (2H, t, *J* = 7.4 Hz, H-3′), *δ*_C_ 27.7 (C-2′), *δ*_H_ 2.03 (2H, m, H-2′); and one oxygenated methyl group at *δ*_C_ 52.1 (4′-OCH_3_) and *δ*_H_ 3.64 (3H, s, 4′-OCH_3_). The above-mentioned resonances corresponded to four indices of unsaturation, indicating the requirement of one more ring in compound **1**. The elucidation of the ^1^H–^1^H COSY correlations identified two separated proton spin systems for the corresponding fragments H-3–H-4–H-5 and H_2_-1′–H_2_-2′–H_2_-3′. Diagnostic HMBC interactions (Figure 2) of H-4/H_2_-1′ to C-2/C-5 readily indicated the presence of a 1*H*-pyrrol-1-yl ring in compound **1**. HMBC cross-peaks from both H-3 and H_2_-7 to C-2 and C-6 unambiguously assigned the hydroxyacetyl group on C-2. Furthermore, the HMBC correlations between H_2_-1′/C-2′ and C-3′; H_2_-2′/C-1′, C-3′ and C-4′; and OCH_3_-4′/C-4′ successfully evidenced the side chain of methyl butanoate connected to the *N*-atom, establishing a characteristic pyrrole alkaloid similar to 4-[5-(2-hydroxymethyl-1-carbonyl)-1*H*-pyrrol-1-yl]butanoic acid from edible mushroom *Lentinula edodes* [13]. Thus, the chemical construction of compound **1** was identified as depicted (Figure 1).

(−)-Loliolide ethyl ether (**2**) was afforded as colourless oil with the molecular formula C_13_H_20_O_3_ in accordance with its ion peak of *m*/*z* 225.1481 [M+H]^+^ (calcd for C_13_H_21_O_3_, 225.1485) recorded using the positive HRESIMS, suggesting four indices of hydrogen deficiency. The ^1^H NMR (Table 1) and ^13^C NMR (Table 1), combining the HSQC and HMBC correlations of compound **2** (Figure 1), disclosed one ester carbonyl at *δ*_C_ 174.4 (C-8); two olefinic carbons (one protonated) at *δ*_C_ 185.7 (C-6), *δ*_C_ 113.3 (C-7) and *δ*_H_ 5.75 (1H, s, H-7); two quaternary carbons (one oxygenated) at *δ*_C_ 89.0 (C-5) and *δ*_C_ 37.2 (C-1); one oxygenated methine at *δ*_C_ 67.3 (C-3) and *δ*_H_ 4.22 (1H, m, H-3); three methylene units (one oxygenated) at *δ*_C_ 58.3 (C-1′), *δ*_H_ 3.61 (2H, q, *J* = 7.1 Hz, H-1′), *δ*_C_ 48.0 (C-2), *δ*_H_ 1.53 (1H, dd, *J* = 14.4, 3.7 Hz, H-2*α*), *δ*_H_ 1.99 (1H, ddd, *J* = 14.4, 3.1, 2.3 Hz, H-2*β*), *δ*_C_ 46.4 (C-4), *δ*_H_ 1.75 (1H, overlapped, H-4*α*) and *δ*_H_ 2.42 (1H, dt, *J* = 13.4, 2.3 Hz, H-4*β*); and four methyl groups at *δ*_C_ 31.0 (C-10), *δ*_H_ 1.28 (3H, s, H-10), *δ*_C_ 27.4 (C-11), *δ*_H_ 1.76 (3H, s, H-11), *δ*_C_ 27.0 (C-9), *δ*_H_ 1.47 (3H, s, H-9), *δ*_C_ 18.4 (C-2′) and *δ*_H_ 1.18 (3H, t, *J* = 7.1 Hz, H-2′). The aforementioned data occupied two degrees of unsaturation, requiring compound **2** to possess another two rings. The ^1^H–^1^H COSY interaction analysis resulted in the identification of two independent proton spin systems for the relevant fragments of H_2_-2–H-3–H_2_-4 and H_2_-1′–H_3_-2′. Integrated NMR data (Table 1) and conclusive HMBC (Figure 2) cross-peaks of H_2_-2/C-3, C-4 and C-6; H-3/C-1, C-2, C-4 and C-5; H_3_-9 (10)/C-1, C-2, C-6 and C-10 (9); and H_3_-11/C-4, C-5 and C-6 indicated that compound **2** owned a six-membered ring bearing two methyl groups at C-1, one methyl group at C-5 and an oxygenated substituent at C-3. The HMBC interactions (Figure 2) of H-7/C-5, C-6 and C-8 disclosed the existence of one α,β-unsaturated lactone ring fused at C-5 and C-6. Except for an additional ethoxy group confirmed using HRESIMS and ^1^H–^1^H COSY spectra, the virtually superimposable NMR data to (−)-loliolide (**3**) readily evidenced the ethoxy residue at C-3 despite the absence of obvious HMBC correlations. The gross structure for compound **2** (Figure 2) was consequently constructed as an ethyl ether of (−)-loliolide (**3**) [14], with the principal difference of 3-OEt in compound **2** instead of 3-OH in **3**.

**Figure 3:**
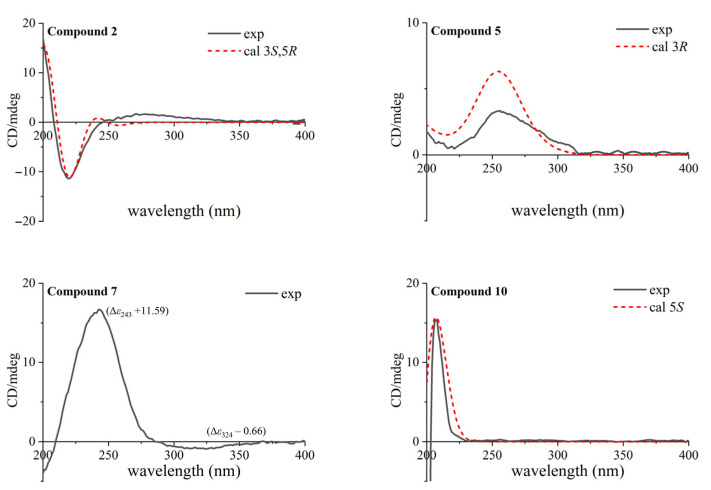
Experimental and calculated ECD spectra of compounds **2**, **5**, **7** and **10**.

The chemical shift of 4.22 (1H, m, H-3), combining its coupling constants around 3.7 Hz with adjacent H_2_-2 and H_2_-4, suggested an equatorial oxymethine proton with *α*-disposition for H-3. The cross-peaks (Figure 2) of H-2*α*/H-3, H-3/H-4*α*, H-4*α*/H-2*α* and H-2*α*/H_3_-10 disclosed *α*-orientations for these protons, which were situated on the same side. Meanwhile, the NOESY correlations of H_3_-11/H-2*β*, H_3_-11/H-4*β* and H_3_-11/H_3_-9 revealed *β*-orientations for those protons, which were located on the opposite side. The ECD spectrum calculated for the 3*S*,5*R* absolute configuration of compound **2** (Figure 3) matched its experimental data, thereby establishing the stereochemical structure above.

Furthermore, nine known isolates were obtained from *Euphorbia esula* for the first time, and their structures were identified to be (−)-loliolide (**3**) [14], loliolide acetate (**4**) [15], (3*R*)-3-hydroxy-*β*-ionone (**5**) [16], (3*S*,5*R*,6*S*,7*E*)-5,6-epoxy-3-hydroxy-7-megastigmen-9-one (**6**) [17], blumenol A (vomifoliol) (**7**) [18], (+)-dehydrovomifoliol (**8**) [19], inotopyrrole (**9**) [20], (5*S*)-5-hydroxy-3,4-dimethyl-5-pentylfuran-2(5*H*)-one (**10**) [21], and chakyunglupulin A (**11**) [22]. The above compounds, except **3**, **6** and **8**, were firstly reported from the genus *Euphorbia*, with their absolute configurations established using experimental and theoretical ECD spectra (Figure 3) for compounds **5**, **7** and **10**. Moreover, the CD extreme values of compound **7** were in better agreement with the (6*S*,9*R*)-megastigmane derivative vomifoliol (blumenol A) (Δ*ε*_240_ +11.9, Δ*ε*_318_ −0.65) [23,24], rather than its (6*S*,9*S*)-epimer corchoionol C (Δ*ε*_242_ +4.1, Δ*ε*_307_ −1.4) [25,26], between which almost indistinguishable NMR signals were reported to display.

### 2.2. Anti-Inflammatory Activity Evaluation for Compounds ***1**–**11***

**Figure 4:**
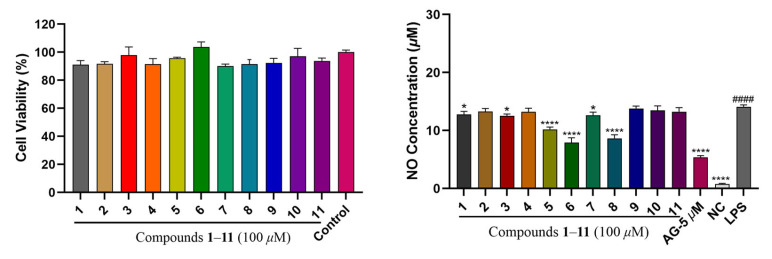
Cell viability and intracellular NO evaluation of compounds **1**–**11** at 100 μM for the anti-inflammatory assay. AG (andrographolide, 5 μM) represented for the positive control. ^####^ *p* < 0.0001 versus the NC (negative control); * *p* < 0.05 and **** *p* < 0.0001 versus the LPS (model control).

To evaluate the anti-inflammatory activities for compounds **1**–**11**, the inhibition levels of NO (nitric oxide) release in LPS (lipopolysaccharide)-stimulated Raw264.7 macrophages were measured in this study, with a positive control of andrographolide (AG, 5 μM). The findings showed that compounds **1** (12.77 ± 0.42 μM), **3** (12.52 ± 0.26 μM), **5** (10.19 ± 0.33 μM), **6** (7.91 ± 0.67 μM), **7** (12.63 ± 0.44 μM) and **8** (8.64 ± 0.50 μM) (Figure 4) exhibited some inhibition on NO production at 100 μM, suggesting anti-inflammatory potential.

### 2.3. Cytotoxicity Evaluation for Compounds ***1**–**11***

Cytotoxic activities for compounds **1**–**11** were determined using an MTT assay on three human cancer cell lines (HT-29, HeLa and MCF-7) at a concentration of 50 μM, with DOX (doxorubicin) employed for the positive control. Unfortunately, none of the aforementioned compounds exhibited significant cytotoxicity.

## 3. Materials and Methods

### 3.1. General Experimental Procedures

See Table S1.

### 3.2. Plant Material

Aerial parts of *Euphorbia esula* (Euphorbiaceae) were gathered in Yangikurgan, Namangan, Republic of Uzbekistan (41°11′14″ N, 71°44′0″ E) on 29 May 2021. The extraction of the research object was performed by Dr. Nurmirza Begmatov from the Acad. S. Yu. Yunusov Institute of the Chemistry of Plant Substances. Taxonomic identification of the plant sample was carried out by Dr. Orzimat Turdimatovich Turginov from the Laboratory of Uzbekistan Flora at the Institute of Botany, with the voucher specimens (TASH00282123–6) deposited there.

### 3.3. Extraction and Separation

After air-drying and pulverizing, the aboveground parts of *Euphorbia esula* (10 kg) were extracted with 80% alcohol (6 × 50 L) at room temperature. Following vacuum evaporation, a crude extract (1165 g) was obtained, dispersed in distilled water (6 L) and divided using ethyl acetate (4 × 6 L) to result in the EtOAc fraction (101 g), which was passed over CC (silica gel column chromatography) with gradient elution of petroleum ether–ethyl acetate (ranging from 1:0–0:1) to yield 8 fractions (1–8). Fr.2 (5.4 g) was separated using RP-18 CC with the eluent MeOH–H_2_O (1:9–1:0) to afford 13 subfractions. Fr.2-3 (47.2 mg) was further performed using semi-PHPLC (ACN–H_2_O, 25:75 *v*/*v*, 3 mL/min) and acquired compound **3** (2.5 mg, *t*_R_ = 20.7 min). Fr.2-5 (39.0 mg) was loaded into semi-PHPLC through the elution of ACN–H_2_O (62:38 *v*/*v*, 3 mL/min) to give compound **4** (1.5 mg, *t*_R_ 24.2 min). Purification of Fr.2-6 (31.0 mg) using semi-PHPLC with the mobile phase ACN–H_2_O (30:70 *v*/*v*, 3 mL/min) was performed to receive compound **9** (1.9 mg, *t*_R_ 49.1 min). Then, Fr.2-7 (42.3 mg) was applied to semi-PHPLC using ACN–H_2_O (45:55 *v*/*v*, 3 mL/min) as the eluent to furnish compound **10** (9.0 mg, *t*_R_ 19.8 min).

Similarly, Fr.4 (11.6 g) was isolated using RP-18 CC carrying MeOH–H_2_O (1:9–1:0) as the mobile phase to generate 20 subfractions. Fr.4-11 (50.7 mg) was further purified via semi-PHPLC with elution of ACN–H_2_O (33:67 *v*/*v*, 3 mL/min) and gained compound **5** (6.3 mg, *t*_R_ 16.4 min).

Likewise, further isolation of Fr.5 (20.1 g) was performed via RP-18 CC, using gradient MeOH–H_2_O (5:95–1:0) to produce 19 subfractions. Fr.5-8 (44.8 mg) was injected into semi-PHPLC and eluted with ACN–H_2_O (14:86 *v*/*v*, 3 mL/min) for the harvest of compound **8** (1.3 mg, *t*_R_ 12.3 min). Fr.5-9 (168.0 mg) was purified using injection into semi-PHPLC with elution of ACN–H_2_O (35:65 *v*/*v*, 3 mL/min), giving compounds **2** (3.5 mg, *t*_R_ 19.7 min) and **6** (3.0 mg, *t*_R_ 23.5 min).

In the same way, the subsequent isolation of Fr.7 (11.6 g) was accomplished via RP-18 CC. A mobile phase composed of MeOH and H_2_O with a gradient ratio ranging from 5:95 to 1:0 was implemented, resulting in the isolation of 7 subfractions. Fr.7-2 (240.3 mg) was passed through semi-PHPLC with ACN–H_2_O (18:82 *v*/*v*, 3 mL/min) as the eluent to obtain compounds **7** (11.6 mg, *t*_R_ 12.1 min), **11** (6.3 mg, *t*_R_ 16.8 min) and **1** (7.9 mg, *t*_R_ 26.5 min).

#### 3.3.1. Methyl 4-[2-(2-Hydroxyacetyl)-1*H*-pyrrol-1-yl]butanoate (**1**)

Colourless oil; UV (MeOH) *λ*_max_ (log *ε*) 288.4 (3.98) nm; IR (KBr) *ν*_max_: 3450, 2956, 2916, 2869, 2841, 2360, 2342, 1733, 1652, 1457, 1379, 946, 747 cm^–1^; ^1^H and ^13^C NMR data, see Table 1; (+)-HRESIMS *m*/*z* 226.1070 [M+H]^+^ (calcd for C_11_H_16_NO_4_, 226.1074)

#### 3.3.2. (−)-Loliolide Ethyl Ether (**2**)

Colourless oil; [*α*]^30^_D_–42 (*c* 0.1, MeOH); UV (MeOH) *λ*_max_ (log *ε*) 200.0 (4.10) nm; ECD (MeOH) *λ*_max_ (Δ*ε*) 220 (−1.54), 271 (+0.23) nm; IR (KBr) *ν*_max_: 3435, 2957, 2919, 1732, 1622, 1456, 1376, 1264, 964 cm^–1^; ^1^H and ^13^C NMR data, see Table 1; (+)-HRESIMS *m*/*z* 225.1481 [M+H]^+^ (calcd for C_13_H_21_O_3_, 225.1485)

#### 3.3.3. (−)-Loliolide (**3**)

White crystals (MeOH–H_2_O); [*α*]^31^_D_–93 (*c* 0.1, MeOH); (+)-HRESIMS *m*/*z* 197.1169 [M+H]^+^ (calcd for C_11_H_17_O_3_, 197.1172); ^1^H NMR (600 MHz, CD_3_OD, δ, ppm): 5.75 (1H, s, H-7), 4.22 (1H, m, H-3), 2.42 (1H, dt, *J* = 13.4, 2.7 Hz, H-4*β*), 2.00 (1H, dt, *J* = 14.4, 2.7 Hz, H-2*β*), 1.77 (3H, s, H-11), 1.75 (1H, overlapped, H-4*α*), 1.53 (1H, dd, *J* = 14.4, 3.7 Hz, H-2*α*), 1.47 (3H, s, H-9), 1.28 (3H, s, H-10); ^13^C NMR (150 MHz, CD_3_OD, δ, ppm): 185.7 (C-6), 174.4 (C-8), 113.3 (C-7), 89.0 (C-5), 67.3 (C-3), 48.0 (C-2), 46.4 (C-4), 37.2 (C-1), 31.0 (C-10), 27.4 (C-11), 27.0(C-9) [14].

#### 3.3.4. Loliolide Acetate (**4**)

Colourless oil; [*α*]^30^_D_–60.1 (*c* 0.05, MeOH); (+)-HRESIMS *m*/*z* 239.1276 [M+H]^+^ (calcd for C_13_H_19_O_4_, 239.1278); ^1^H NMR (600 MHz, CD_3_OD, δ, ppm): 5.82 (1H, s, H-7), 5.24 (1H, tt, *J* = 4.1, 2.8 Hz, H-3), 2.51 (1H, dt, *J* = 14.2, 2.5 Hz, H-4*β*), 2.10 (3H, s, H-2′), 2.07 (1H, dt, *J* = 15.1, 2.5 Hz, H-2*β*), 1.87 (1H, ddq, *J* = 14.2, 4.2, 0.9 Hz, H-4*α*), 1.73 (3H, d, *J* = 0.9 Hz, H-11), 1.65 (1H, dd, *J* = 15.1, 4.0 Hz, H-2*α*), 1.43 (3H, s, H-9), 1.31 (3H, s, H-10); ^13^C NMR (150 MHz, CD_3_OD, δ, ppm): 184.0 (C-6), 173.9 (C-8), 171.9 (C-1′), 114.1 (C-7), 87.9 (C-5), 70.2 (C-3), 45.0 (C-2), 43.7 (C-4), 36.9 (C-1), 30.8 (C-10), 27.0 (C-11), 26.4 (C-9), 21.2 (C-2′) [15].

#### 3.3.5. (3*R*)-3-Hydroxy-*β*-ionone (**5**)

Colourless oil; [*α*]^29^_D_–77 (*c* 0.1, MeOH); (+)-HRESIMS *m*/*z* 209.1532 [M+H]^+^ (calcd for C_13_H_21_O_2_, 209.1536); ^1^H NMR (600 MHz, CD_3_OD, δ, ppm): 7.33 (1H, d, *J* = 16.4 Hz, H-7), 6.14 (1H, d, *J* = 16.4 Hz, H-8), 3.93 (1H, m, H-3), 2.41 (1H, dd, *J* = 17.9, 5.9 Hz, H-4eq), 2.31 (3H, s, H-10), 2.07 (1H, dd, *J* = 17.9, 9.6 Hz, H-4ax), 1.80 (3H, s, H-13), 1.78 (1H, m, H-2eq), 1.46 (1H, t, *J* = 12.1 Hz, H-2ax), 1.15 (3H, s, H-12), 1.12 (3H, s, H-11); ^13^C NMR (150 MHz, CD_3_OD, δ, ppm): 201.2 (C-9), 144.5 (C-7), 136.9 (C-6), 134.3 (C-8), 133.2 (C-5), 64.9 (C-3), 49.3 (C-2), 43.5 (C-4), 37.8 (C-1), 30.6 (C-11), 28.8 (C-12), 27.2 (C-10), 21.7 (C-13) [16].

#### 3.3.6. (3*S*,5*R*,6*S*,7*E*)-5,6-Epoxy-3-hydroxy-7-megastigmen-9-one (**6**)

Colourless oil; [*α*]^30^_D_–100 (*c* 0.1, MeOH); (+)-HRESIMS *m*/*z* 225.1481 [M+H]^+^ (calcd for C_13_H_21_O_3_, 225.1485); ^1^H NMR (400 MHz, CDCl_3_, δ, ppm): 7.03 (1H, d, *J* = 15.6 Hz, H-7), 6.29 (1H, d, *J* = 15.6 Hz, H-8), 3.92 (1H, m, H-3), 2.39 (1H, ddd, *J* = 14.5, 5.2, 1.7 Hz, H-4*α*), 2.28 (3H, s, H-10), 1.65 (1H, dd, *J* = 14.5, 8.7 Hz, H-4*β*), 1.62 (1H, m, H-2*α*), 1.36 (1H, m, H-2*β*), 1.19 (3H, s, H-13), 1.19 (3H, s, H-11), 0.97 (3H, s, H-12); ^13^C NMR (100 MHz, CDCl_3_, δ, ppm): 197.6 (C-9), 142.5 (C-7), 132.8 (C-8), 69.6 (C-6), 67.4 (C-5), 64.2 (C-3), 46.8 (C-4), 40.7 (C-2), 35.3 (C-1), 29.5 (C-11), 28.4 (C-10), 25.1 (C-12), 20.0 (C-13) [17].

#### 3.3.7. Blumenol A (Vomifoliol) (**7**)

White amorphous powder; [*α*]^30^_D_+109 (*c* 0.1, MeOH); CD (MeOH) λ_max_ (Δ*ε*) 243 (+11.59) 324 (−0.66) nm; (+)-HRESIMS *m*/*z* 207.1377 [M+H–H_2_O]^+^ (calcd for C_13_H_19_O_2_, 207.1380); ^1^H NMR (600 MHz, CDCl_3_, δ, ppm): 5.90 (1H, br s, H-4), 5.87 (1H, dd, *J* = 15.7, 5.4 Hz, H-8), 5.79 (1H, d, *J* = 15.7 Hz, H-7), 4.41 (1H, m, H-9), 2.45 (1H, d, *J* = 17.0 Hz, H-2*β*), 2.24 (1H, d, *J* = 17.0 Hz, H-2*α*), 1.90 (3H, d, *J* = 1.4 Hz, H-13), 1.30 (3H, d, *J* = 6.4 Hz, H-10), 1.08 (3H, s, H-12), 1.00 (3H, s, H-11); ^13^C NMR (150 MHz, CDCl_3_, δ, ppm): 198.1 (C-3), 162.8 (C-5), 135.9 (C-8), 129.1 (C-7), 127.0 (C-4), 79.2 (C-6), 68.1 (C-9), 49.9 (C-2), 41.3 (C-1), 24.2 (C-11), 23.9 (C-10), 23.0 (C-12), 19.0 (C-13) [18].

#### 3.3.8. (+)-Dehydrovomifoliol (**8**)

Yellowish oil; [*α*]^30^_D_+94.6 (*c* 0.05, MeOH); (+)-HRESIMS *m*/*z* 223.1325 [M+H]^+^ (calcd for C_13_H_19_O_3_, 223.1329); ^1^H NMR (600 MHz, CD_3_OD, δ, ppm): 7.00 (1H, d, *J* = 15.8 Hz, H-7), 6.44 (1H, d, *J* = 15.8 Hz, H-8), 5.94 (1H, s, H-4), 2.61 (1H, d, *J* = 17.2 Hz, H-2a), 2.31 (3H, s, H-10), 2.28 (1H, d, *J* = 17.2 Hz, H-2b), 1.90 (3H, d, *J* = 1.3 Hz, H-13), 1.07 (3H, s, H-11), 1.02 (3H, s, H-12); ^13^C NMR (100 MHz, CD_3_OD, δ, ppm): 200.7 (C-9), 200.4 (C-3), 164.7 (C-5), 148.3 (C-7), 131.7 (C-8), 128.0 (C-4), 80.0 (C-6), 50.5 (C-2), 42.7 (C-1), 27.6 (C-10), 24.7 (C-12), 23.5 (C-11), 19.2 (C-13) [19].

#### 3.3.9. Inotopyrrole (**9**)

Colourless oil; (+)-HRESIMS *m*/*z* 230.1174 [M+H]^+^ (calcd for C_14_H_16_NO_2_, 230.1176); ^1^H NMR (600 MHz, CD_3_OD, δ, ppm): 9.47 (1H, s, H-6), 7.25 (2H, m, H-5′, H-7′), 7.21 (1H, m, H-6′), 7.13 (2H, m, H-4′, H-8′), 7.02 (1H, d, *J* = 4.0 Hz, H-3), 6.21 (1H, d, *J* = 4.0 Hz, H-4), 4.54 (2H, t, *J* = 7.4 Hz, H-1′), 4.28 (2H, s, H-7), 3.02 (2H, t, *J* = 7.4 Hz, H-2′); ^13^C NMR (150 MHz, CD_3_OD, δ, ppm): 180.9 (C-6), 144.8 (C-5), 139.9 (C-3′), 133.4 (C-2), 130.1 (C-4′, C-8′), 129.5 (C-5′, C-7′), 127.6 (C-6′), 126.5 (C-3), 111.2 (C-4), 56.4 (C-7), 48.7 (C-1′), 38.7 (C-2′) [20].

#### 3.3.10. (5*S*)-5-Hydroxy-3,4-dimethyl-5-pentylfuran-2(5*H*)-one (**10**)

Colourless oil; [*α*]^30^_D_–50 (*c* 0.1, MeOH); (+)-HRESIMS *m*/*z* 199.1326 [M+H]^+^ (calcd for C_11_H_19_O_3_, 199.1329); ^1^H NMR (400 MHz, CD_3_OD, δ, ppm): 1.90 (3H, d, *J* = 1.4 Hz, H-7), 1.83 (2H, m, H-1′), 1.76 (d, *J* = 1.4 Hz, 3H, H-6), 1.29 (4H, m, H-2′, 3′), 1.16 (2H, m, H-4′), 0.87 (3H, t, *J* = 7.0 Hz, H-5′); ^13^C NMR (100 MHz, CD_3_OD, δ, ppm): 174.5 (C-2), 160.3 (C-4), 125.7 (C-3), 109.4 (C-5), 36.9 (C-1′), 32.8 (C-3′), 23.8 (C-2′), 23.5 (C-4′), 14.3 (C-5′), 10.8 (C-7), 8.2 (C-6) [21].

#### 3.3.11. Chakyunglupulin A (**11**)

Amorphous powder; [*α*]^30^_D_–57 (*c* 0.1, MeOH); (+)-HRESIMS *m*/*z* 197.1171 [M+H−H_2_O]^+^ (calcd for C_11_H_17_O_3_, 197.1172); ^1^H NMR (600 MHz, CD_3_OD, δ, ppm): 5.77 (1H, s, H-2), 4.10 (1H, tt, *J* = 11.4, 4.2 Hz, H-6), 2.47 (1H, ddd, *J* = 11.8, 4.2, 2.2 Hz, H-5*β*), 2.01 (1H, ddd, *J* = 13.0, 4.3, 2.2 Hz, H-7*β*), 1.59 (3H, d, *J* = 0.9 Hz, H-11), 1.42 (1H, td, *J* = 11.6, 0.9 Hz, H-5*α*), 1.31 (3H, s, H-10), 1.29 (1H, overlapped, H-7*α*), 1.29 (3H, s, H-9); ^13^C NMR (150 MHz, CD_3_OD, δ, ppm): 183.9 (C-1), 174.0 (C-3), 113.7 (C-2), 88.6 (C-4), 65.3 (C-6), 50.7 (C-7), 48.9 (C-5), 36.2 (C-8), 30.3 (C-10), 25.8 (C-11), 25.3 (C-9) [22].

### 3.4. Calculations of ECD Spectra

Energy computations and geometric optimizations for the conformations were conducted using TmoleX 3.3 software, adopting the TDDFT (time-dependent density functional theory) approach at the b3-lyp/m3-TZVP level. Followed by obtaining the stable conformations, ECD (electronic circular dichroism) calculations were carried out with the above-described TDDFT method and level to afford the theoretical ECD spectra [27].

### 3.5. Anti-Inflammatory Effect Assay

#### 3.5.1. Cell Culture

Sourced from the BeNa Culture Collection, Raw264.7 macrophages were dispersed and then transferred to an incubator for cultivation, which was maintained at 37 °C with a 5% CO_2_ atmosphere. After 24 h of incubation, when the cell density reached 80%, 3 mL of culture medium was added. The medium was gently pipetted to prepare a cell suspension and then subculture it at a 1:5 ratio for continued cultivation.

#### 3.5.2. Cell Viability Test Using the CCK-8 Method

The CCK-8 assay was employed to test cell viability [28]. Raw264.7 macrophages at a density of 8 × 10^3^ cells/100 μL were seeded into 96-well plates and cultured overnight in a 37 °C incubator with 5% CO_2_. Next, the cells were treated for 1 h with either sample solutions of varying concentrations or positive control AG (andrographolide, HY-N0191) at 5 µM in DMSO. Following this, the cells were further induced using 1 μg/mL lipopolysaccharide (LPS, L4391, Sigma-Aldrich, St Louis, MO, USA) for 16 h. After removing the supernatant, each well was added 100 μL of CCK-8 solution (C0038, Beyotime, Shanghai, China) and further incubated for 1–2 h. A microplate reader was then used to measure the absorbance at 450 nm for the calculation of cell viability relative to the control cells.

#### 3.5.3. NO Release Evaluation by the Griess Method

Anti-inflammatory effects for all compounds were assessed by measuring their capacities to inhibit the intracellular NO release in RAW 264.7 cells stimulated via LPS, applying the Griess approach with andrographolide serving as a positive control. Samples were added to the cells and incubated for 1 h, then further incubated with 1 μg/mL LPS for an additional 16 h. The collected supernatant and standards at concentrations of 0, 1, 2, 5, 10, 20, 40, 60 and 100 μM were added to 96-well plates. Then, 50 μL of the Nitric Oxide Assay Kit reagent (S0021M, Beyotime, Shanghai, China) was added to each well of the plates and gently shaken at room temperature. Subsequently, a microplate reader was utilized to measure the absorbance at 540 nm and calculate the intracellular NO content according to the standard curve.

### 3.6. Cytotoxic Effect Assay

#### 3.6.1. Cell Culture

Three human cancer cell lines of HT-29, HeLa and MCF-7 were obtained from Cell Bank of the Chinese Academy of Sciences and cultured in a medium consisting of DMEM, 10% FBS (fetal bovine serum) and 1% penicillin–streptomycin within an incubator of 37 °C and 5% CO_2_.

#### 3.6.2. Cell Growth Inhibition by MTT Assay

The cytotoxic capacities for isolated compounds against the above three cell lines were evaluated utilizing the MTT assay [29]. Settled in 96-well plates, the cells were hatched overnight at 37 °C and treated with samples of varying concentrations for an additional 48 h. Replaced with MTT solution (5 mg/mL), the culture medium was then maintained at 37 °C for 3–4 h. Next, a microplate reader was applied to measure the absorbance at 570 nm and calculate the cell inhibition rate accordingly.

## 4. Conclusions

This chemical study brought about two previously undescribed compounds (**1**–**2**), together with nine reported ones (**3**–**11**) from the aboveground organs of *Euphorbia esula* growing in Uzbekistan. Their structures were established on the basis of comprehensive spectroscopic data, with corresponding absolute configurations determined according to experimental and computational ECD spectra data. The resulting isolates demonstrated diverse structural frameworks including two pyrrole alkaloids (**1**, **9**), seven norisoprenoids (**2**–**8**), one furanone (**10**) and another unusual cyclooct-2-enone (**11**). Previous studies on *Euphorbia* species have shown that, apart from the well-known diterpenoids, reports on alkaloids and norisoprenoids in this genus are relatively scarce. In this study, the discovery of the aforementioned derivatives from *E. esula* further enriched the structural diversity of the genus *Euphorbia*. Additionally, bioactivity tests showed that some compounds (**1**, **3**, **5**, **6**, **7** and **8**) displayed certain anti-inflammatory properties by suppressing the NO release in RAW 264.7 macrophages induced by LPS. The findings of this investigation could benefit further in-depth research in future.

## Figures and Tables

**Table 1:** ^1^H (600 MHz) and ^13^C NMR (150 MHz) data of compounds **1**–**2** in CD_3_OD (*δ* in ppm) ^a^.

No.	1		2	
	*δ*_H_ (*J* in Hz)	*δ*_C_	*δ*_H_ (*J* in Hz)	*δ*_C_
1	-	-	-	37.2, C
2	-	128.1, C	1.99, ddd (14.4, 3.1, 2.3), *β* 1.53, dd (14.4, 3.7), *α*	48.0, CH_2_
3	7.07, overlapped	120.5, CH	4.22, m	67.3, CH
4	6.17, dd (3.9, 2.7)	109.7, CH	2.42, dt (13.4, 2.3), *β* 1.75, overlapped, *α*	46.4, CH_2_
5	7.08, overlapped	132.5, CH	-	89.0, C
6	-	189.9, C	-	185.7, C
7	4.63, s	65.4, CH_2_	5.75, s	113.3, CH
8	-	-	-	174.4, C
9	-	-	1.47, s	27.0, CH_3_
10	-	-	1.28, s	31.0, CH_3_
11	-	-	1.76, s	27.4, CH_3_
1′	4.41, t (6.9)	49.5, CH_2_	3.61, q (7.1)	58.3, CH_2_
2′	2.03, m	27.7, CH_2_	1.18, t (7.1)	18.4, CH_3_
3′	2.28, t (7.4)	31.5, CH_2_	-	-
4′	-	175.1, C	-	-
4′-OCH_3_	3.64, s	52.1, CH_3_	-	-

^a^ Assigned on the basis of HSQC and HMBC correlations, with coupling constants in parentheses.

## Data Availability

Data are contained within the article and Supplementary Materials.

## Supplementary Materials

The following supporting information can be downloaded at: https://www.mdpi.com/article/10.3390/plants14182822/s1, Figure S1-1. ^1^H NMR spectrum (CD_3_OD, 600 MHz) of compound **1**; Figure S1-2. ^13^C NMR spectrum (CD_3_OD, 150 MHz) of compound **1**; Figure S1-3. ^1^H-^1^H COSY spectrum (CD_3_OD) of compound **1**; Figure S1-4. HSQC spectrum (CD_3_OD) of compound **1**; Figure S1-5. HMBC spectrum (CD_3_OD) of compound **1**; Figure S1-6. (+)-HRESIMS spectrum of compound **1**; Figure S1-7. UV spectrum (MeOH) of compound **1**; Figure S1-8. IR spectrum of compound **1**; Figure S2-1. ^1^H NMR spectrum (CD_3_OD, 600 MHz) of compound **2**; Figure S2-2. ^13^C NMR spectrum (CD_3_OD, 150 MHz) of compound **2**; Figure S2-3. ^1^H-^1^H COSY spectrum (CD_3_OD) of compound **2**; Figure S2-4. HSQC spectrum (CD_3_OD) of compound **2**; Figure S2-5. HMBC spectrum (CD_3_OD) of compound **2**; Figure S2-6. NOESY spectrum (CD_3_OD) of compound **2**; Figure S2-7. (+)-HRESIMS spectrum of compound **2**; Figure S2-8. UV spectrum (MeOH) of compound **2**; Figure S2-9. IR spectrum of compound **2**; Figure S3-1. ^1^H NMR spectrum (CD_3_OD, 600 MHz) of compound **3**; Figure S3-2. ^13^C NMR spectrum (CD_3_OD, 150 MHz) of compound **3**; Figure S3-3. (+)-HRESIMS spectrum of compound **3**; Figure S4-1. ^1^H NMR spectrum (CD_3_OD, 600 MHz) of compound **4**; Figure S4-2. ^13^C NMR spectrum (CD_3_OD, 150 MHz) of compound **4**; Figure S4-3. (+)-HRESIMS spectrum of compound **4**; Figure S5-1. ^1^H NMR spectrum (CD_3_OD, 600 MHz) of compound **5**; Figure S5-2. ^13^C NMR spectrum (CD_3_OD, 150 MHz) of compound **5**; Figure S5-3. (+)-HRESIMS spectrum of compound **5**; Figure S6-1. ^1^H NMR spectrum (CDCl_3_, 400 MHz) of compound **6**; Figure S6-2. ^13^C NMR spectrum (CDCl_3_, 100 MHz) of compound **6**; Figure S6-3. (+)-HRESIMS spectrum of compound **6**; Figure S7-1. ^1^H NMR spectrum (CDCl_3_, 600 MHz) of compound **7**; Figure S7-2. ^13^C NMR spectrum (CDCl_3_, 150 MHz) of compound **7**; Figure S7-3. (+)-HRESIMS spectrum of compound **7**; Figure S8-1. ^1^H NMR spectrum (CD_3_OD, 600 MHz) of compound **8**; Figure S8-2. ^13^C NMR spectrum (CD_3_OD, 150 MHz) of compound **8**; Figure S8-3. (+)-HRESIMS spectrum of compound **8**; Figure S9-1. ^1^H NMR spectrum (CD_3_OD, 600 MHz) of compound **9**; Figure S9-2. ^13^C NMR spectrum (CD_3_OD, 150 MHz) of compound **9**; Figure S9-3. (+)-HRESIMS spectrum of compound **9**; Figure S10-1. ^1^H NMR spectrum (CD_3_OD, 400 MHz) of compound **10**; Figure S10-2. ^13^C NMR spectrum (CD_3_OD, 100 MHz) of compound **10**; Figure S10-3. (+)-HRESIMS spectrum of compound **10**; Figure S11-1. ^1^H NMR spectrum (CD_3_OD, 600 MHz) of compound **11**; Figure S11-2. ^13^C NMR spectrum (CD_3_OD, 150 MHz) of compound **11**; Figure S11-3. (+)-HRESIMS spectrum of compound **11**. Table S1. Equipment used for analyses.
