# Supplementary material for: Chemical Constituents from Euphorbia esula
Source: Plants (Basel). 2025 Sep 9;14(18):2822. doi: 10.3390/plants14182822 (PMC12473696; doi:10.3390/plants14182822)
Supplement: Supplementary file 1 [file plants-14-02822-s001.zip › plants-3855605-supplementary.pdf]

Supplementary information

Chemical Constituents from *Euphorbia esula*

Defeng Yan [1, 2], Miaomiao Zhang [1, 2], Yuqing Song [1, 2], Liu Liu [1], Nurmirza Begmatov [3], Orzimat Turdimatovich Turginov [4], Bo Zhao [1], Hequn Yang [1] and Guoan Zou[1,2,*]

1 State Key Laboratory Basis of Xinjiang Indigenous Medicinal Plants Resource Utilization, Xinjiang Technical Institute of Physics and Chemistry, Chinese Academy of Sciences, Urumqi, 830011, China; yandefeng21@mails.ucas.ac.cn (D.Y.); zhangmiaomiao20@mails.ucas.ac.cn (M.Z.); songyuqing22@mails.ucas.ac.cn (Y.S.); liuliu@ms.xjb.ac.cn (L.L.); zhaobo@ms.xjb.ac.cn (B.Z.); yanghq@ms.xjb.ac.cn (H.Y.)

2 University of Chinese Academy of Sciences, Beijing, 100049, China

3 Acad. S. Yu. Yunusov Institute of the Chemistry of Plant Substances, Academy of Sciences of the Republic of Uzbekistan, 77, M. Ulugbek str., Tashkent, 100170, Uzbekistan; nurmirza87@yahoo.com

4 Institute of Botany, Academy of Sciences of the Republic of Uzbekistan, Tashkent, 100047, Uzbekistan; orzimat@mail.ru

* Correspondence: gazou@ms.xjb.ac.cn

Supplementary Information Contents

Figure S1. NMR, MS, UV and IR Spectra of Compound 1 .... **1**
Figure S1-1. ^{1}H NMR spectrum (CD_3OD, 600 MHz) of compound **1** 1
Figure S1-2. ^{13}C NMR spectrum (CD_3OD, 150 MHz) of compound **1** 1
Figure S1-3. ^{1}H-^{1}H COSY spectrum (CD_3OD) of compound **1** 2
Figure S1-4. HSQC spectrum (CD_3OD) of compound **1** 2
Figure S1-5. HMBC spectrum (CD_3OD) of compound **1** 3
Figure S1-6. (+)-HRESIMS spectrum of compound **1** 3
Figure S1-7. UV spectrum (MeOH) of compound **1** 4
Figure S1-8. IR spectrum of compound **1** 4
Figure S2. NMR, MS, UV and IR Spectra of Compound 2 .... **5**
Figure S2-1. ^{1}H NMR spectrum (CD_3OD, 600 MHz) of compound **2** 5
Figure S2-2. ^{13}C NMR spectrum (CD_3OD, 150 MHz) of compound **2** 5
Figure S2-3. ^{1}H-^{1}H COSY spectrum (CD_3OD) of compound **2** 6
Figure S2-4. HSQC spectrum (CD_3OD) of compound **2** 6
Figure S2-5. HMBC spectrum (CD_3OD) of compound **2** 7
Figure S2-6. NOESY spectrum (CD_3OD) of compound **2** 7
Figure S2-7. (+)-HRESIMS spectrum of compound **2** 8
Figure S2-8. UV spectrum (MeOH) of compound **2** 8
Figure S2-9. IR spectrumof compound **2** 8
Figure S3. NMR and MS of Compound 3 .... **9**
Figure S3-1. ^{1}H NMR spectrum (CD_3OD, 600 MHz) of compound **3** 9
Figure S3-2. ^{13}C NMR spectrum (CD_3OD, 150 MHz) of compound **3** 9
Figure S3-3. (+)-HRESIMS spectrum of compound **3** 10
Figure S4. NMR and MS of Compound 4 .... **11**
Figure S4-1. ^{1}H NMR spectrum (CD_3OD, 600 MHz) of compound **4** 11
Figure S4-2. ^{13}C NMR spectrum (CD_3OD, 150 MHz) of compound **4** 11
Figure S4-3. (+)-HRESIMS spectrum of compound **4** 12
Figure S5. NMR and MS of Compound 5 .... **13**
Figure S5-1. ^{1}H NMR spectrum (CD_3OD, 600 MHz) of compound **5** 13
Figure S5-2. ^{13}C NMR spectrum (CD_3OD, 150 MHz) of compound **5** 13
Figure S5-3. (+)-HRESIMS spectrum of compound **5** 14
Figure S6. NMR and MS of Compound 6 .... **15**
Figure S6-1. ^{1}H NMR spectrum ($CDCl_3$, 400 MHz) of compound **6** 15
Figure S6-2. ^{13}C NMR spectrum ($CDCl_3$, 100 MHz) of compound **6** 15
Figure S6-3. (+)-HRESIMS spectrum of compound **6** 16
Figure S7. NMR and MS of Compound 7 .... **17**
Figure S7-1. ^{1}H NMR spectrum ($CDCl_3$, 600 MHz) of compound **7** 17
Figure S7-2. ^{13}C NMR spectrum ($CDCl_3$, 150 MHz) of compound **7** 17
Figure S7-3. (+)-HRESIMS spectrum of compound **7** 18
Figure S8. NMR and MS of Compound 8 .... **19**
Figure S8-1. ^{1}H NMR spectrum (CD_3OD, 600 MHz) of compound **8** 19
Figure S8-2. ^{13}C NMR spectrum (CD_3OD, 150 MHz) of compound **8** 19
Figure S8-3. (+)-HRESIMS spectrum of compound **8** 20
Figure S9. NMR and MS of Compound 9 .... **21**
Figure S9-1. ^{1}H NMR spectrum (CD_3OD, 600 MHz) of compound **9** 21
Figure S9-2. ^{13}C NMR spectrum (CD_3OD, 150 MHz) of compound **9** 21
Figure S9-3. (+)-HRESIMS spectrum of compound **9** 22
Figure S10. NMR and MS of Compound 10 .... **23**
Figure S10-1. ^{1}H NMR spectrum (CD_3OD, 400 MHz) of compound **10** 23
Figure S10-2. ^{13}C NMR spectrum (CD_3OD, 100 MHz) of compound **10** 23
Figure S10-3. (+)-HRESIMS spectrum of compound **10** 24
Figure S11. NMR and MS of Compound 11 .... **25**
Figure S11-1. ^{1}H NMR spectrum (CD_3OD, 600 MHz) of compound **11** 25
Figure S11-2. ^{13}C NMR spectrum (CD_3OD, 150 MHz) of compound **11** 25
Figure S11-3. (+)-HRESIMS spectrum of compound **11** 26
Table S1. Equipment used for analyses **27**

Figure S1. NMR, MS, UV and IR Spectra of Compound 1

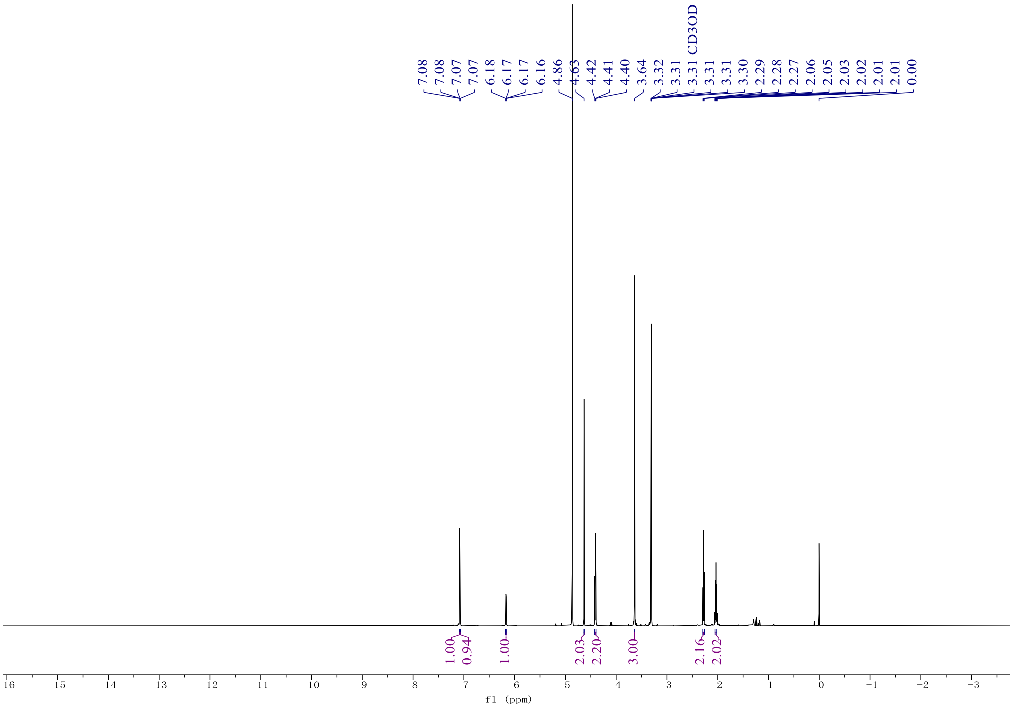

Figure S1-1. ¹H NMR spectrum (CD_3OD, 600 MHz) of compound **1**

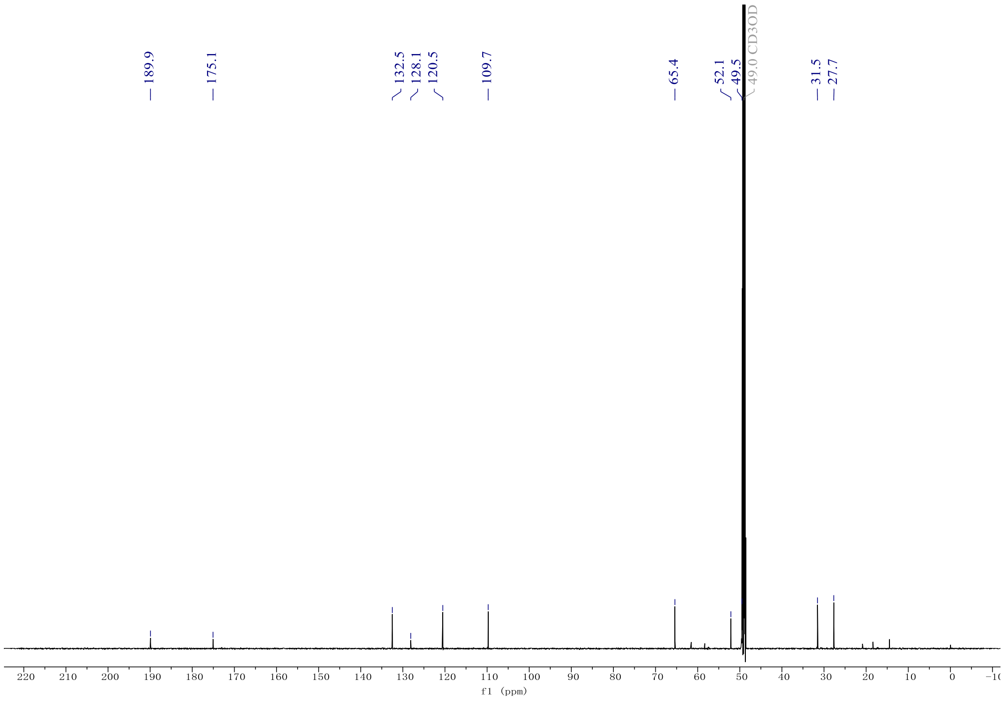

Figure S1-2. ¹³C NMR spectrum (CD_3OD, 150 MHz) of compound **1**

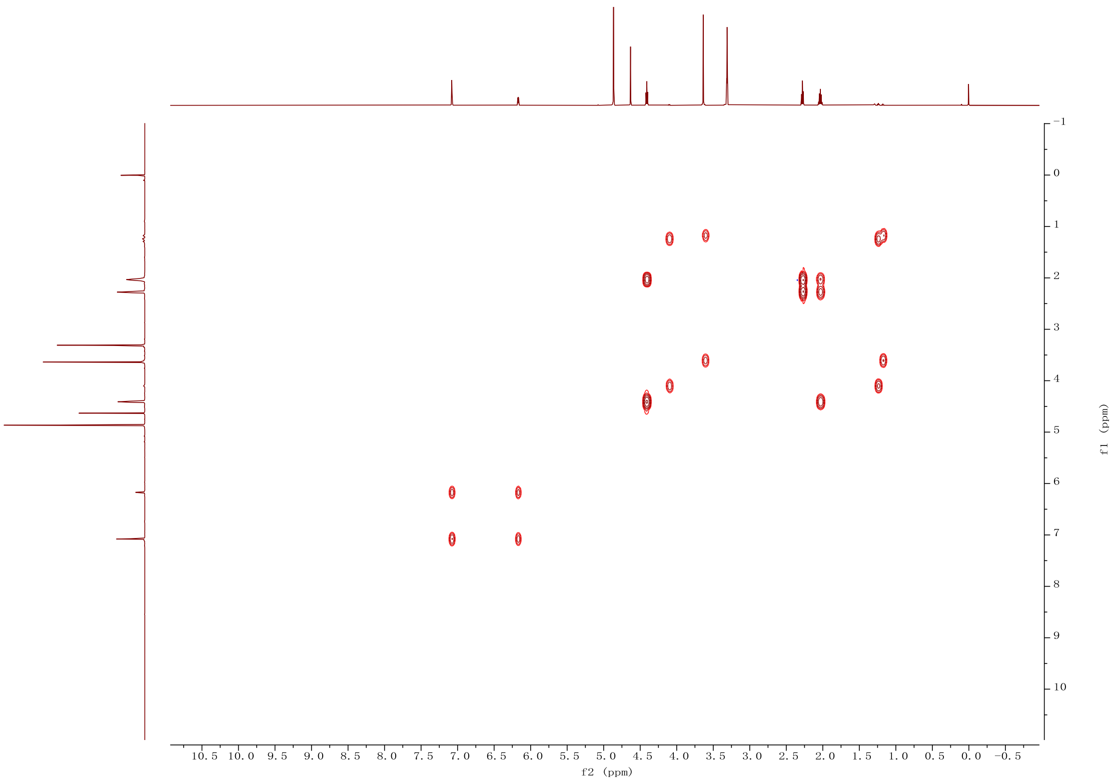

Figure S1-3. ^1H-^1H COSY spectrum (CD_3OD) of compound **1**

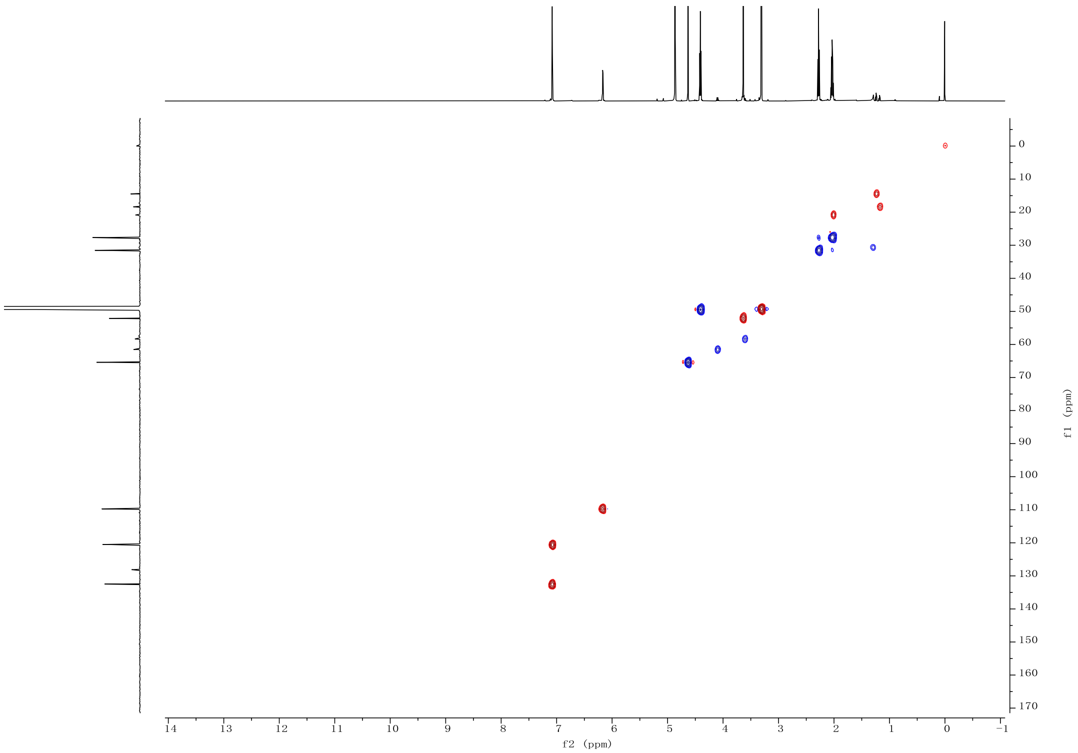

Figure S1-4. HSQC spectrum (CD_3OD) of compound **1**

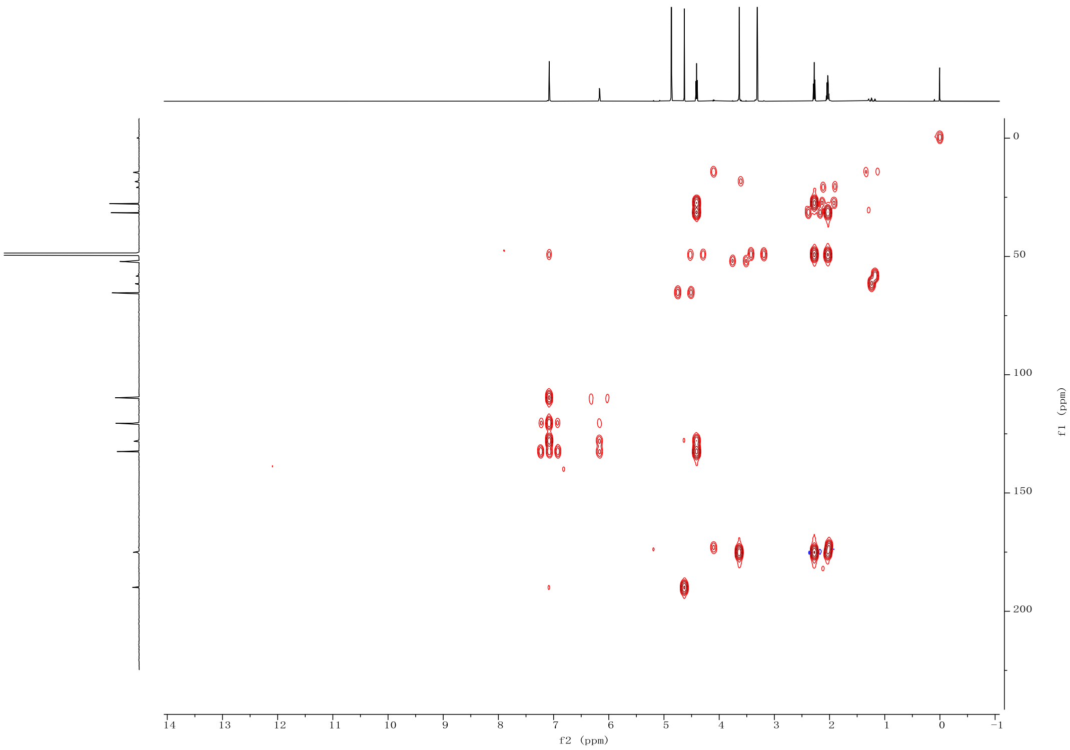

Figure S1-5. HMBC spectrum (CD_3OD) of compound **1**

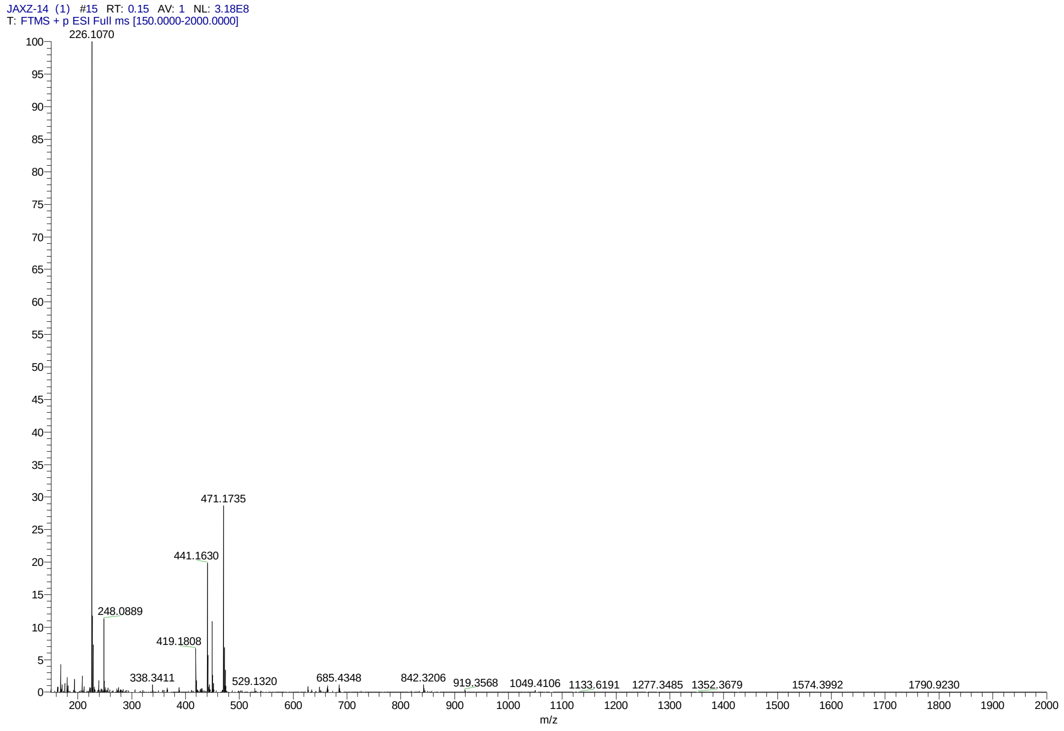

Figure S1-6. (+)-HRESIMS spectrum of compound **1**

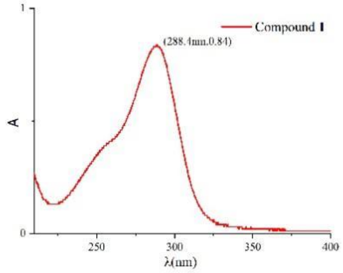

Figure S1-7. UV spectrum (MeOH) of compound **1**

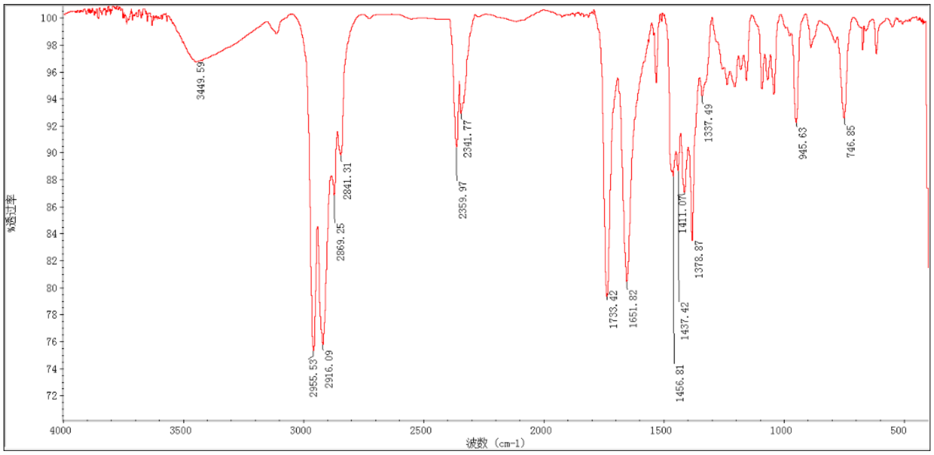

Figure S1-8. IR spectrum of compound **1**

Figure S2. NMR, MS, UV and IR Spectra of Compound 2

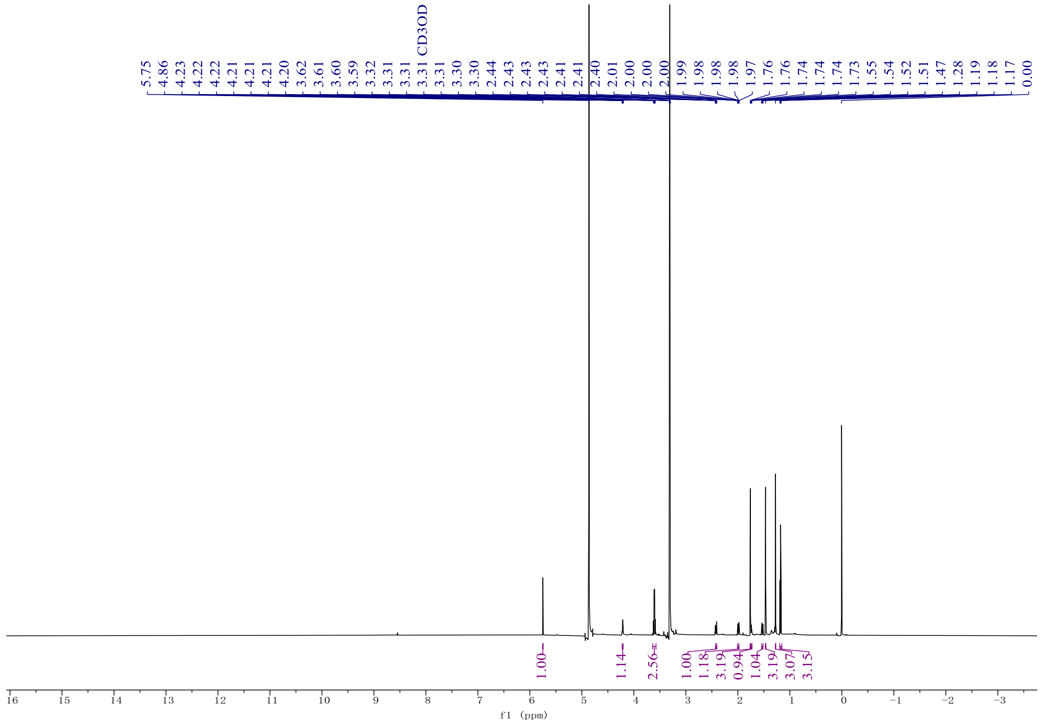

Figure S2-1. ^{1}H NMR spectrum (CD_3OD, 600 MHz) of compound **2**

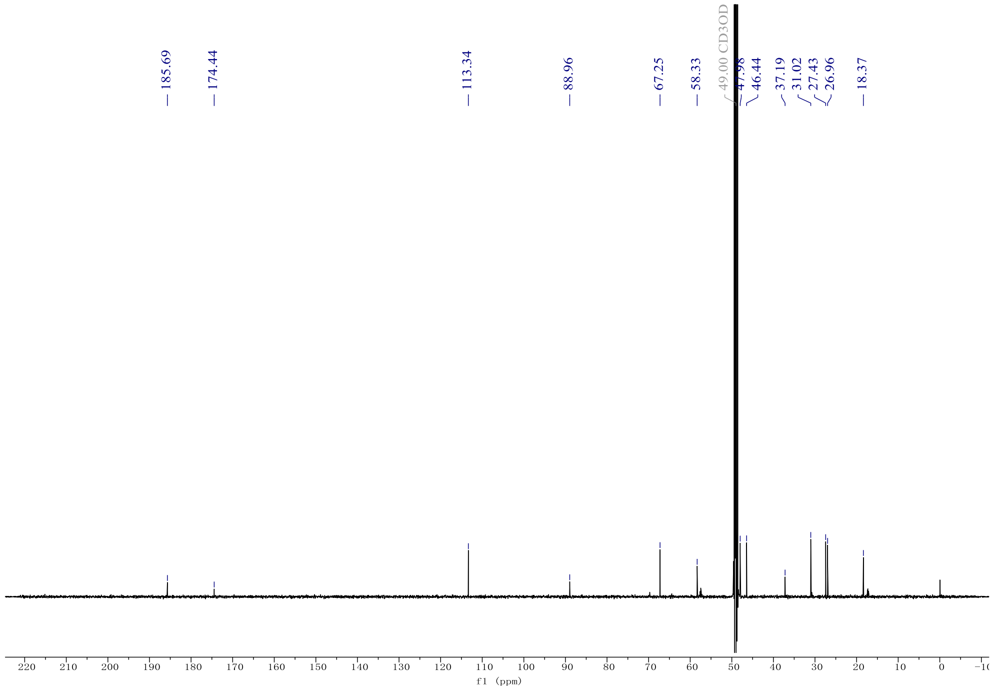

Figure S2-2. ^{13}C NMR spectrum (CD_3OD, 150 MHz) of compound **2**

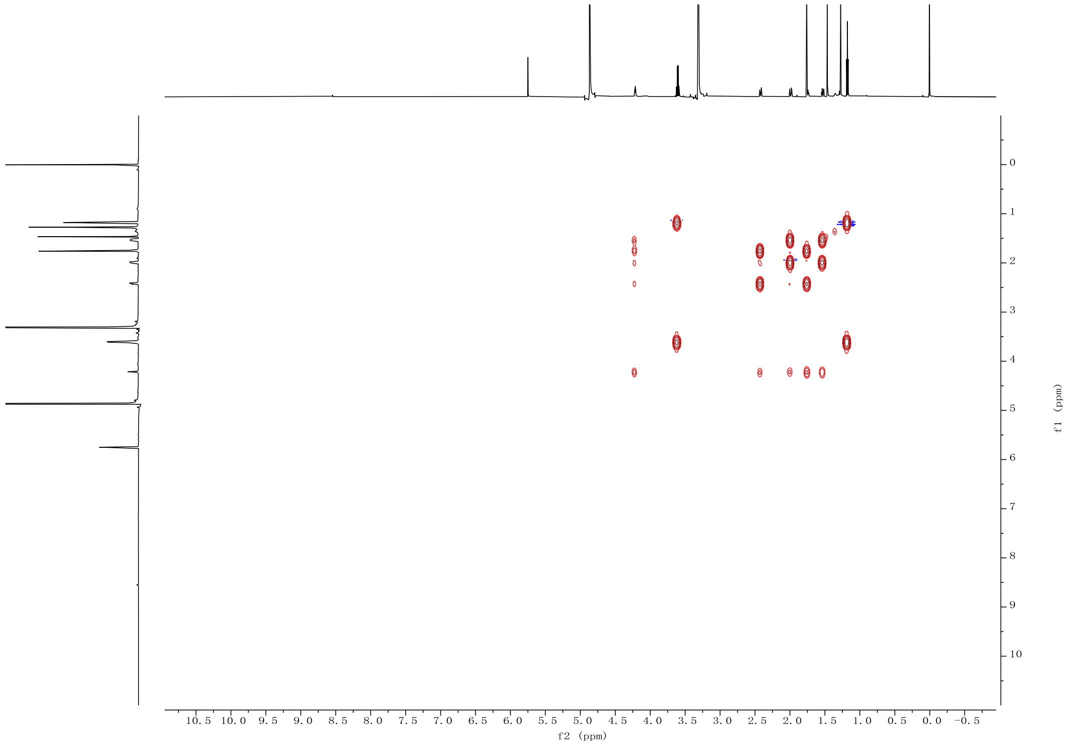

Figure S2-3. ^1H-^1H COSY spectrum (CD_3OD) of compound **2**

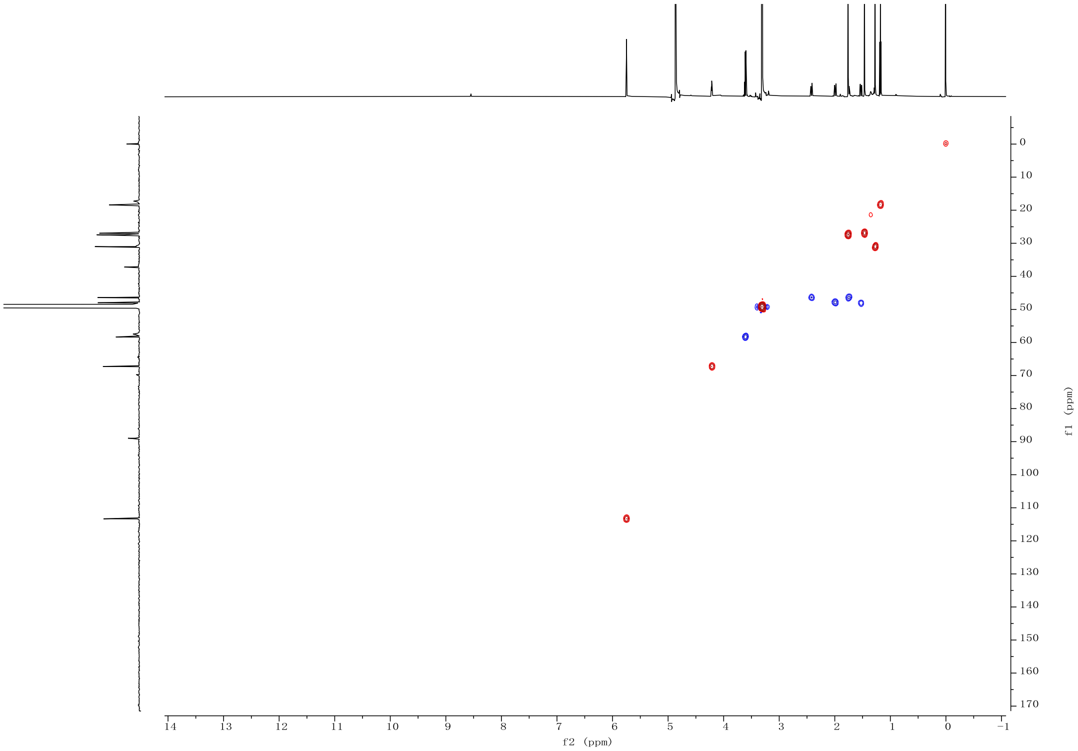

Figure S2-4. HSQC spectrum (CD_3OD) of compound **2**

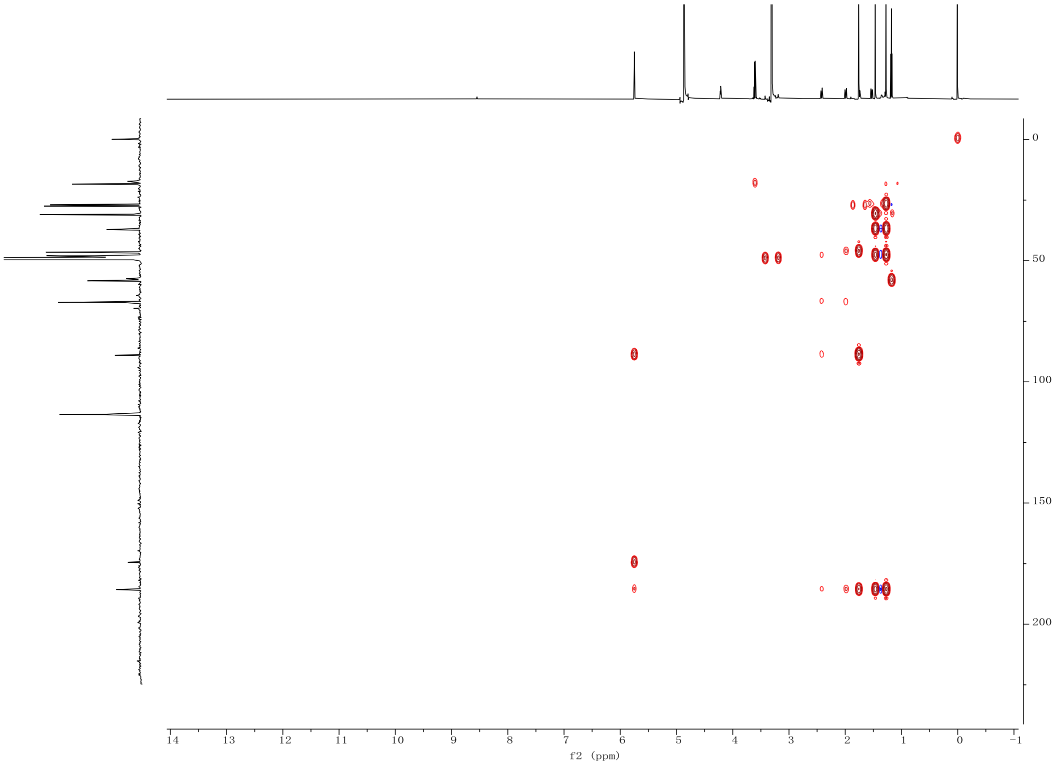

Figure S2-5. HMBC spectrum (CD_3OD) of compound **2**

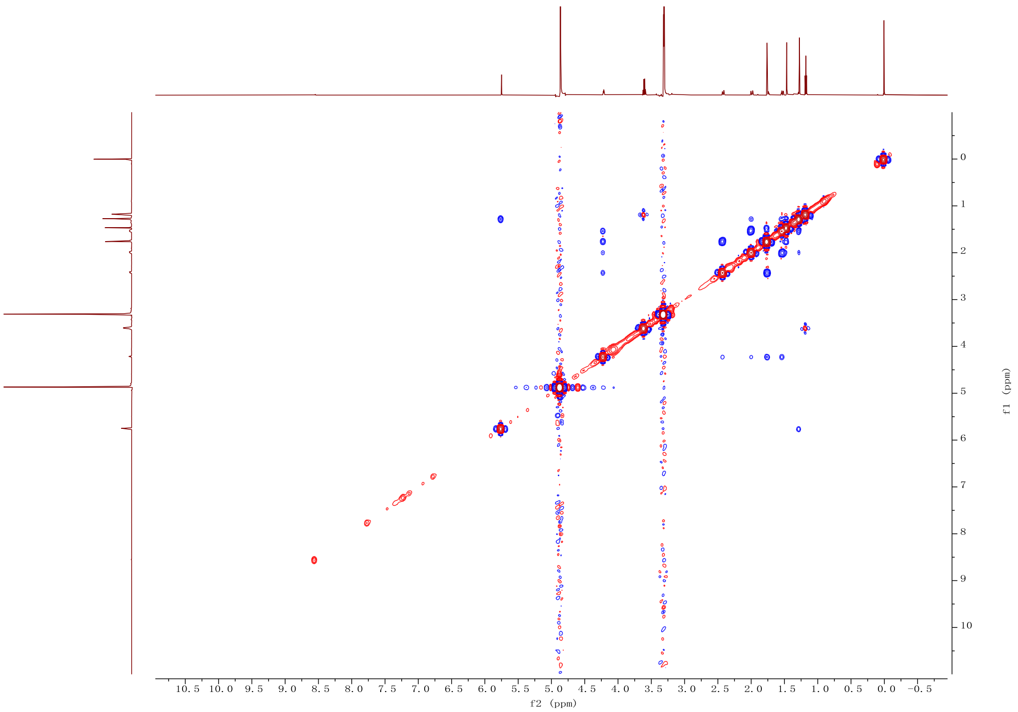

Figure S2-6. NOESY spectrum (CD_3OD) of compound **2**

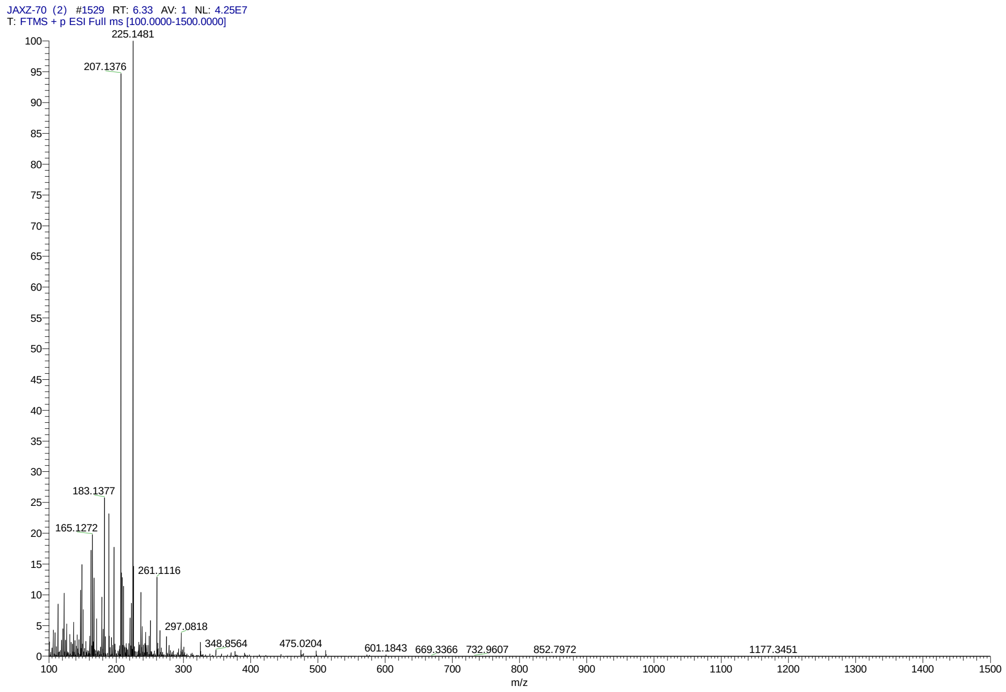

Figure S2-7. (+)-HRESIMS spectrum of compound **2**

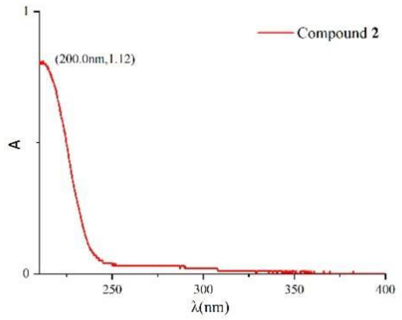

Figure S2-8. UV spectrum (MeOH) of compound **2**

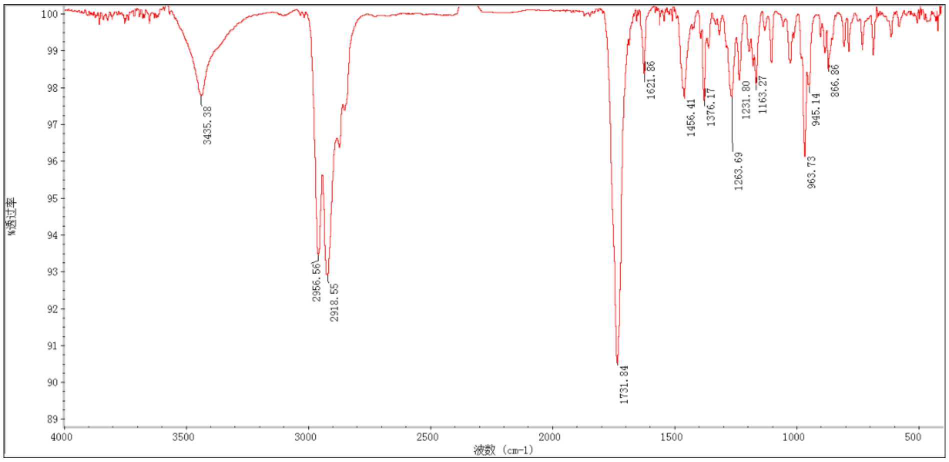

Figure S2-9. IR spectrumof compound **2**

Figure S3. NMR and MS of Compound 3

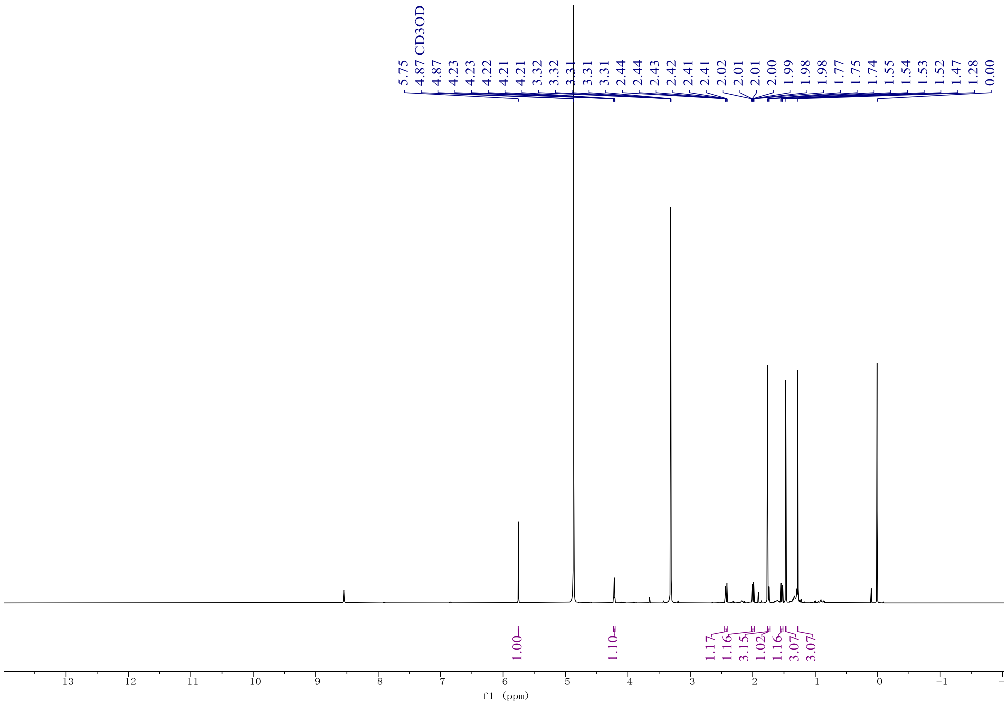

Figure S3-1. ^{1}H NMR spectrum (CD_3OD, 600 MHz) of compound **3**

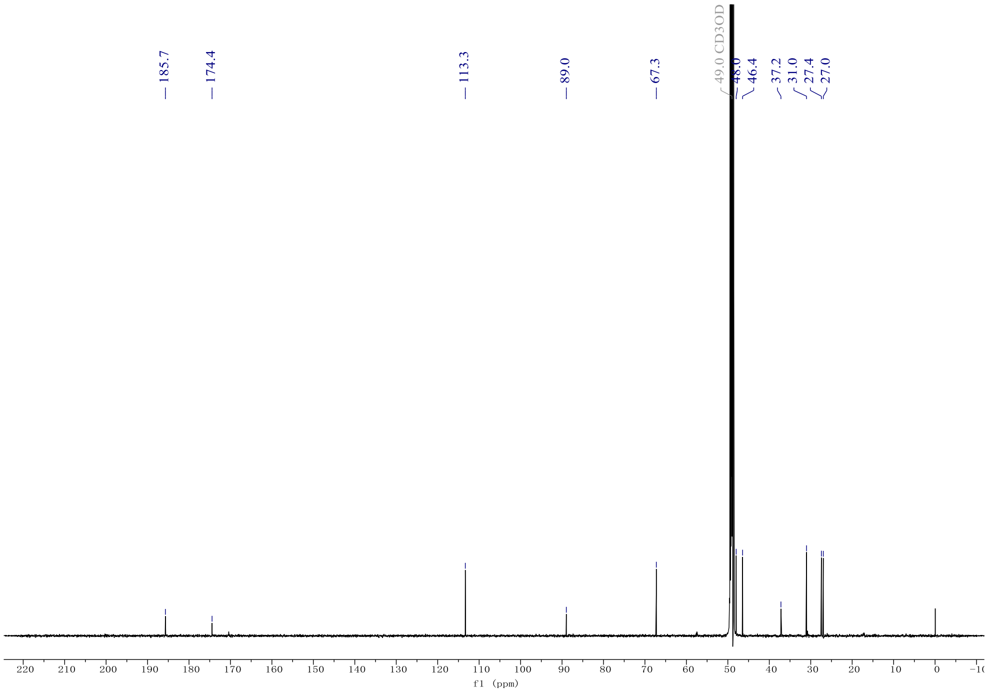

Figure S3-2. ^{13}C NMR spectrum (CD_3OD, 150 MHz) of compound **3**

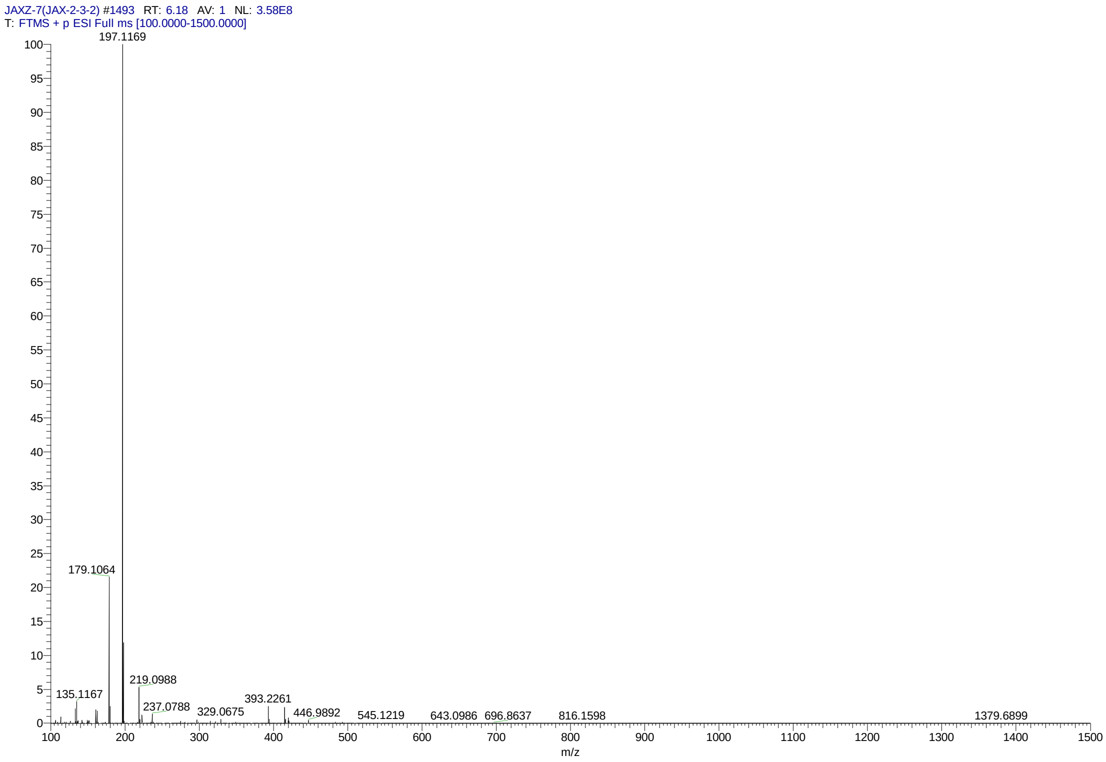

Figure S3-3. (+)-HRESIMS spectrum of compound **3**

Figure S4. NMR and MS of Compound 4

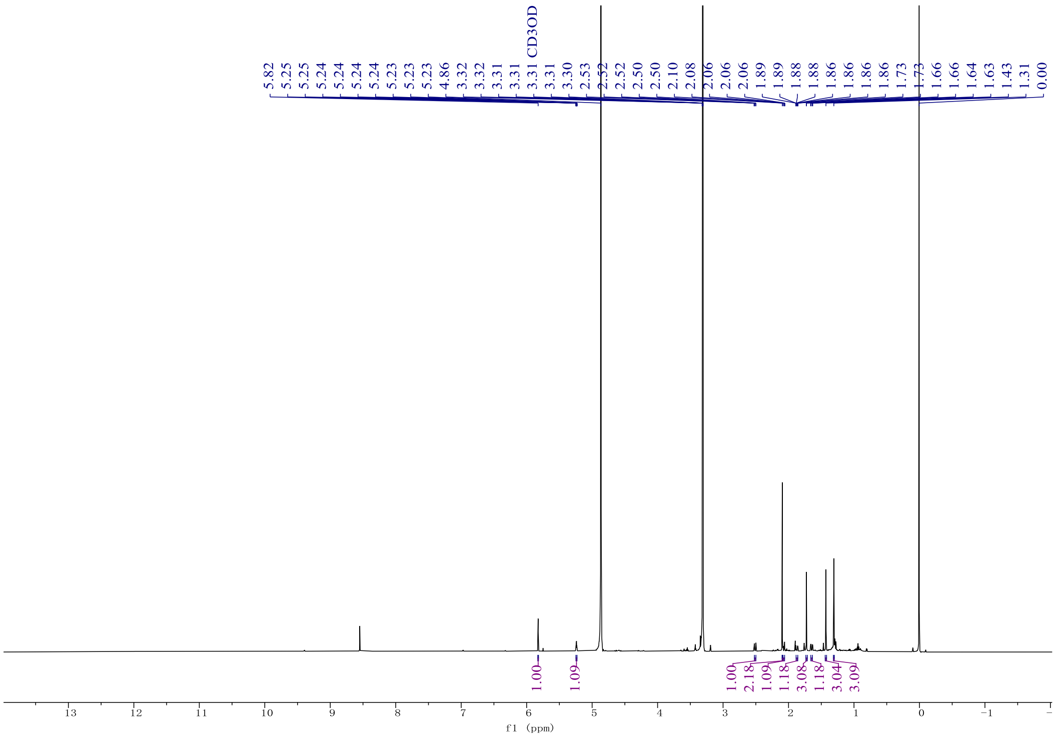

Figure S4-1. ^{1}H NMR spectrum (CD_3OD, 600 MHz) of compound **4**

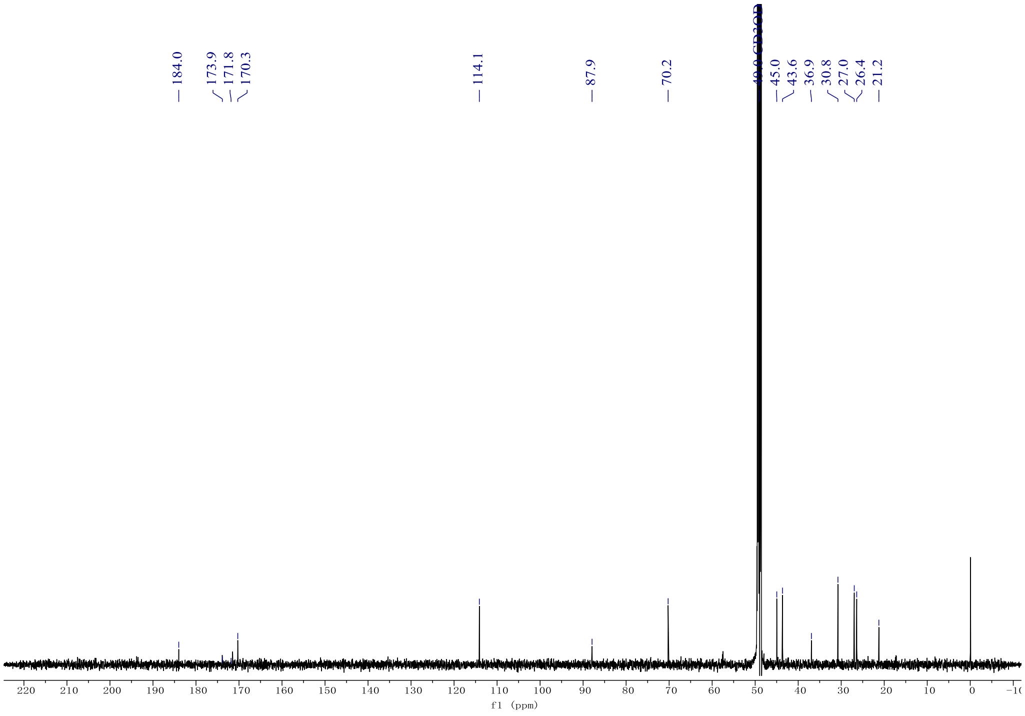

Figure S4-2. ^{13}C NMR spectrum (CD_3OD, 150 MHz) of compound **4**

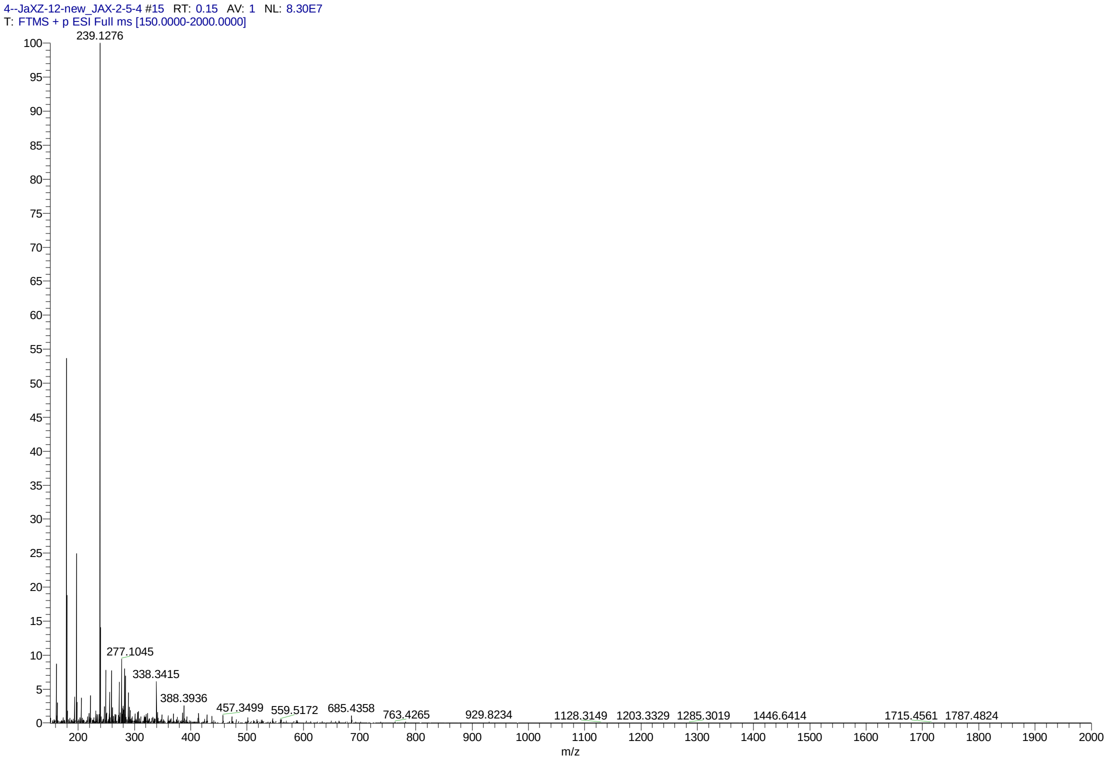

Figure S4-3. (+)-HRESIMS spectrum of compound **4**

Figure S5. NMR and MS of Compound 5

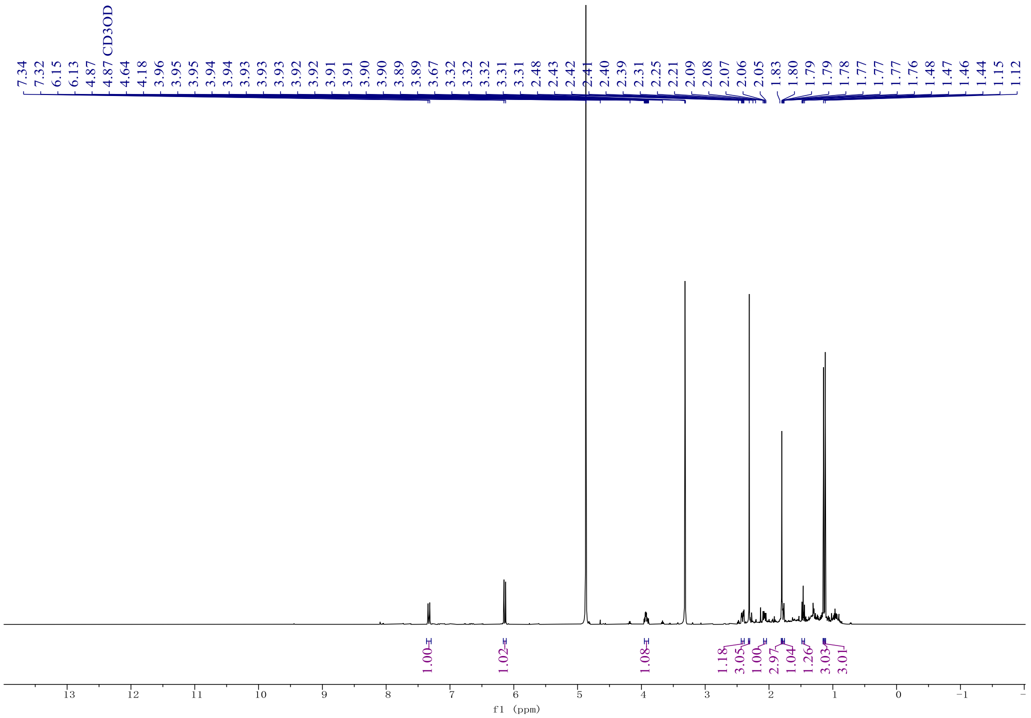

Figure S5-1. ^{1}H NMR spectrum (CD_3OD, 600 MHz) of compound **5**

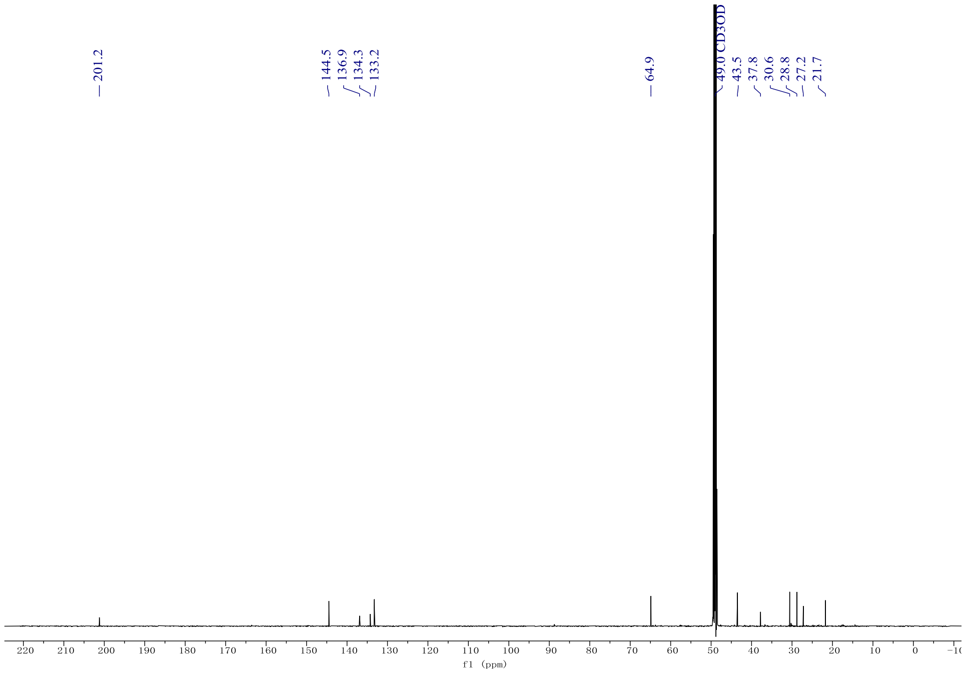

Figure S5-2. ^{13}C NMR spectrum (CD_3OD, 150 MHz) of compound **5**

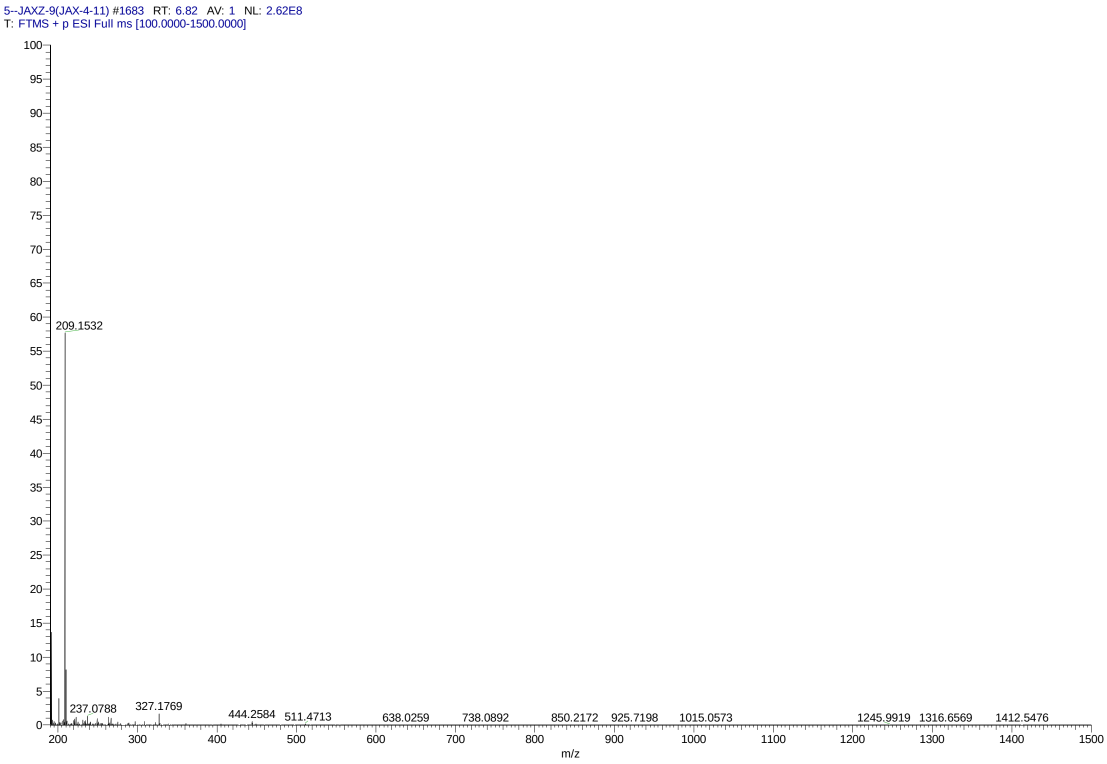

Figure S5-3. (+)-HRESIMS spectrum of compound **5**

Figure S6. NMR and MS of Compound 6

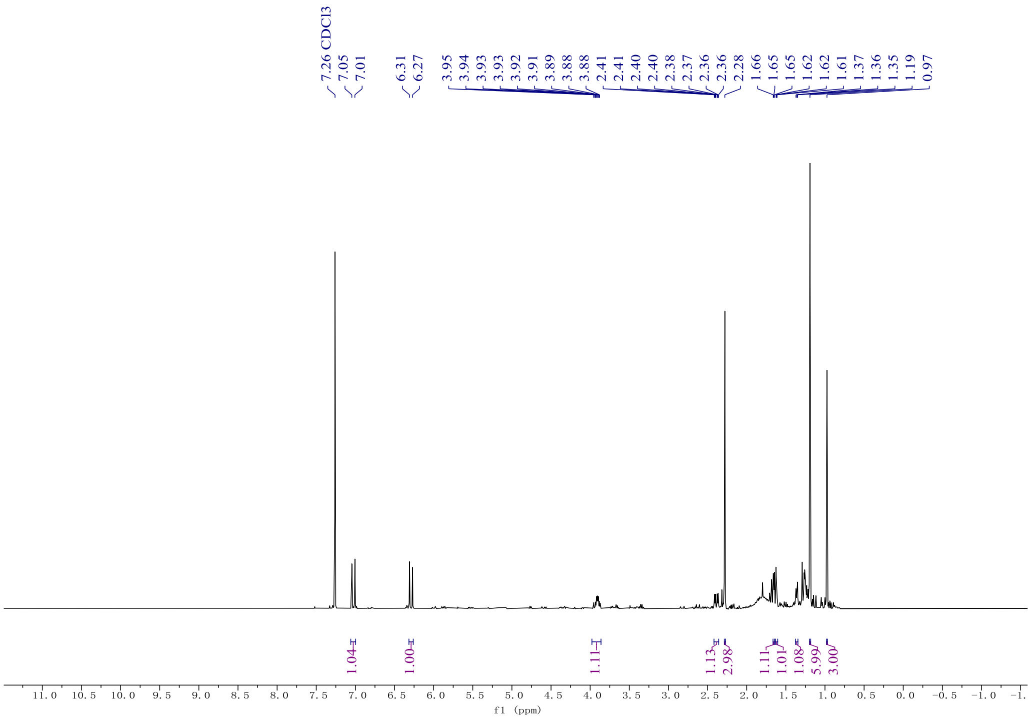

Figure S6-1. ^{1}H NMR spectrum ($CDCl_3$, 400 MHz) of compound **6**

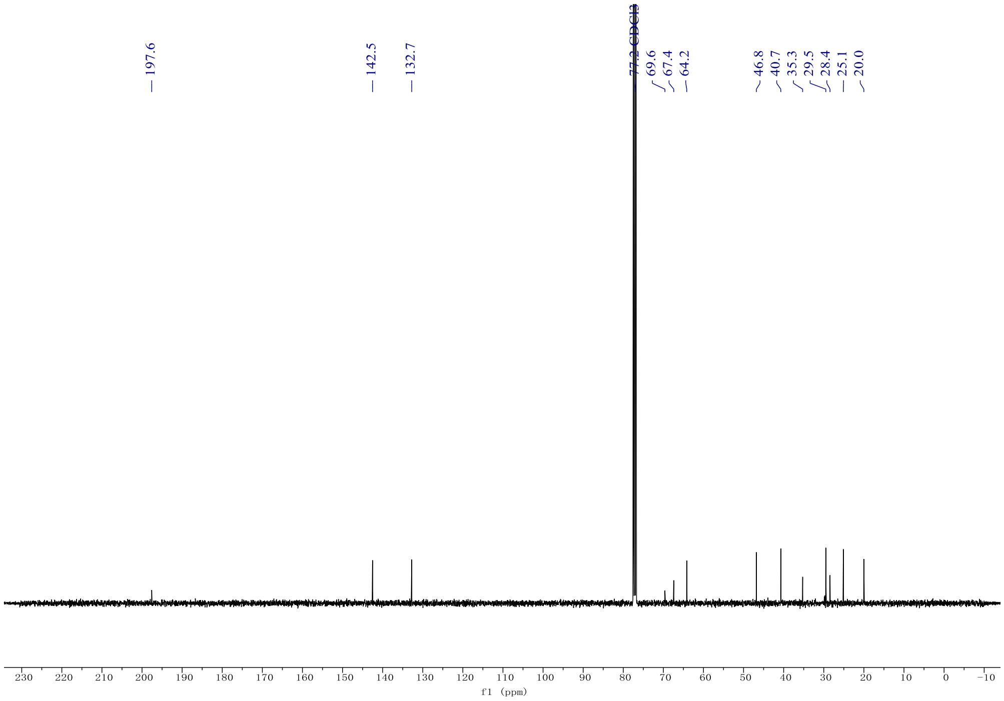

Figure S6-2. ^{13}C NMR spectrum ($CDCl_3$, 100 MHz) of compound **6**

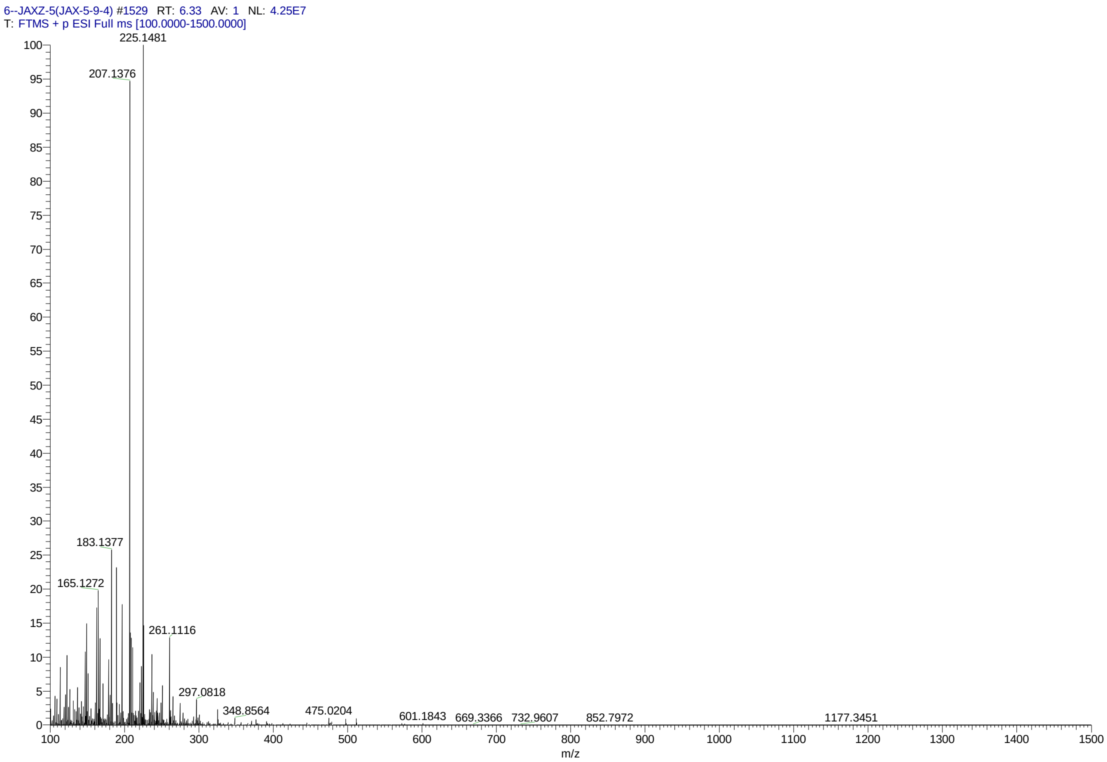

Figure S6-3. (+)-HRESIMS spectrum of compound **6**

Figure S7. NMR and MS of Compound 7

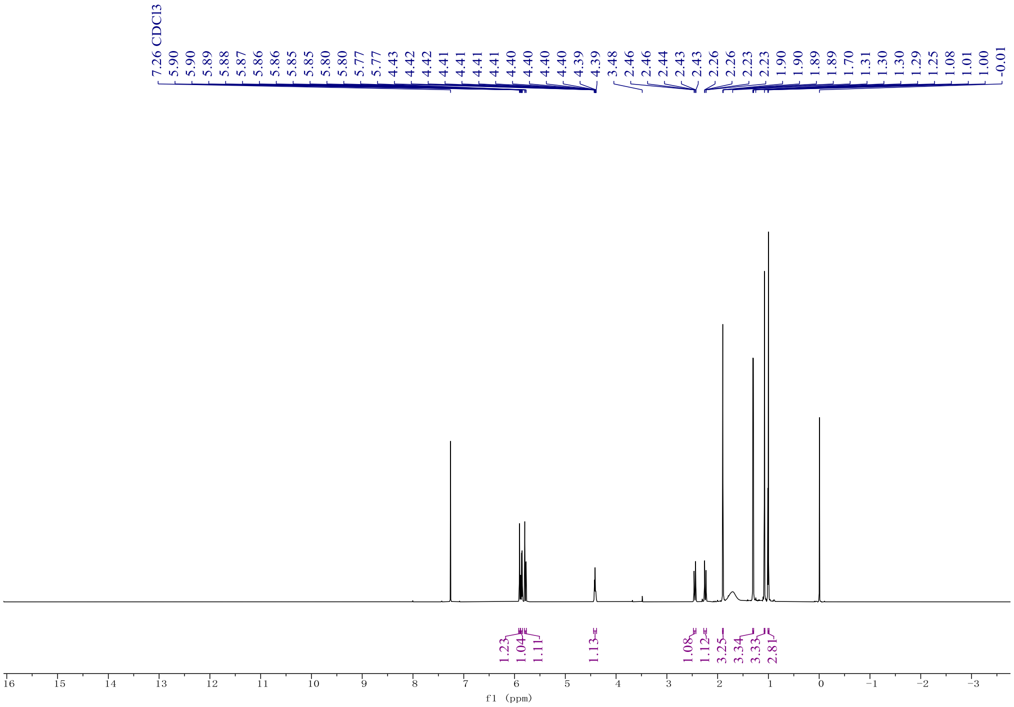

Figure S7-1. ^{1}H NMR spectrum ($CDCl_3$, 600 MHz) of compound **7**

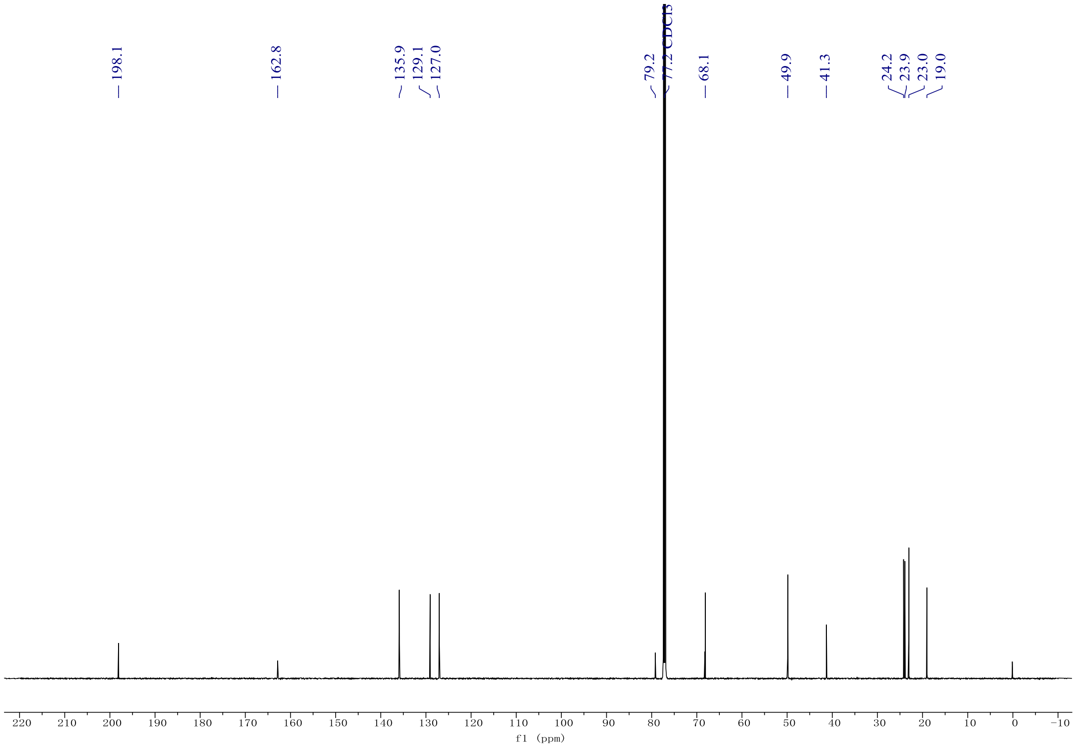

Figure S7-2. ^{13}C NMR spectrum ($CDCl_3$, 150 MHz) of compound **7**

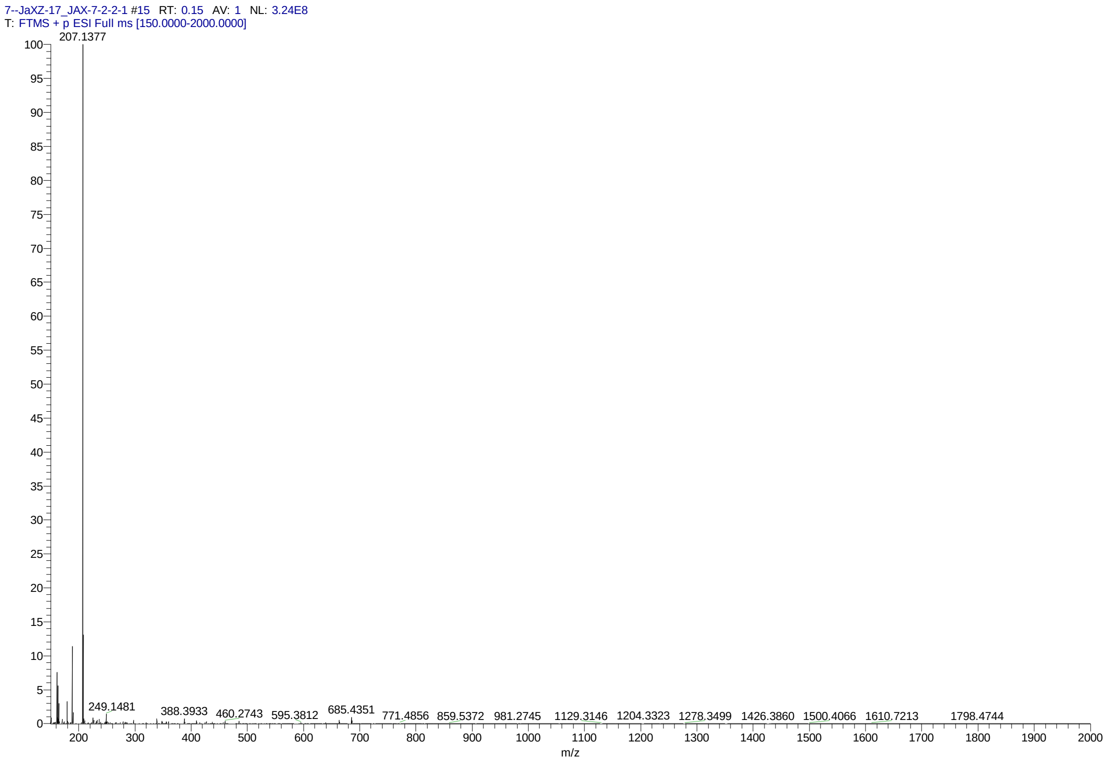

Figure S7-3. (+)-HRESIMS spectrum of compound **7**

Figure S8. NMR and MS of Compound 8

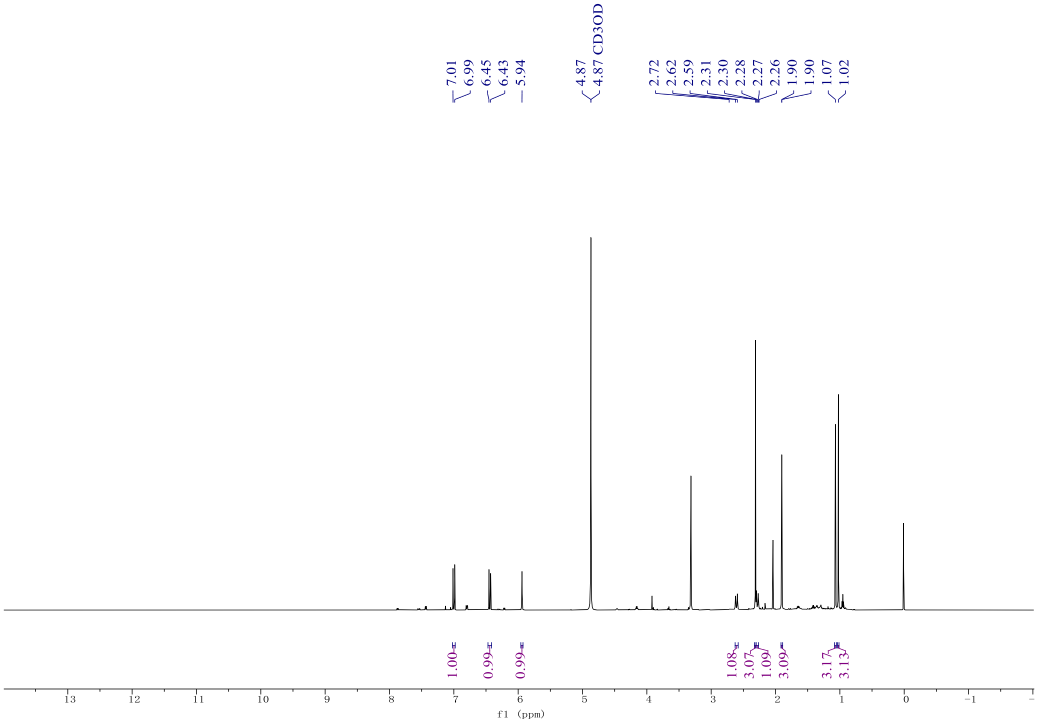

Figure S8-1. ^{1}H NMR spectrum (CD_3OD, 600 MHz) of compound **8**

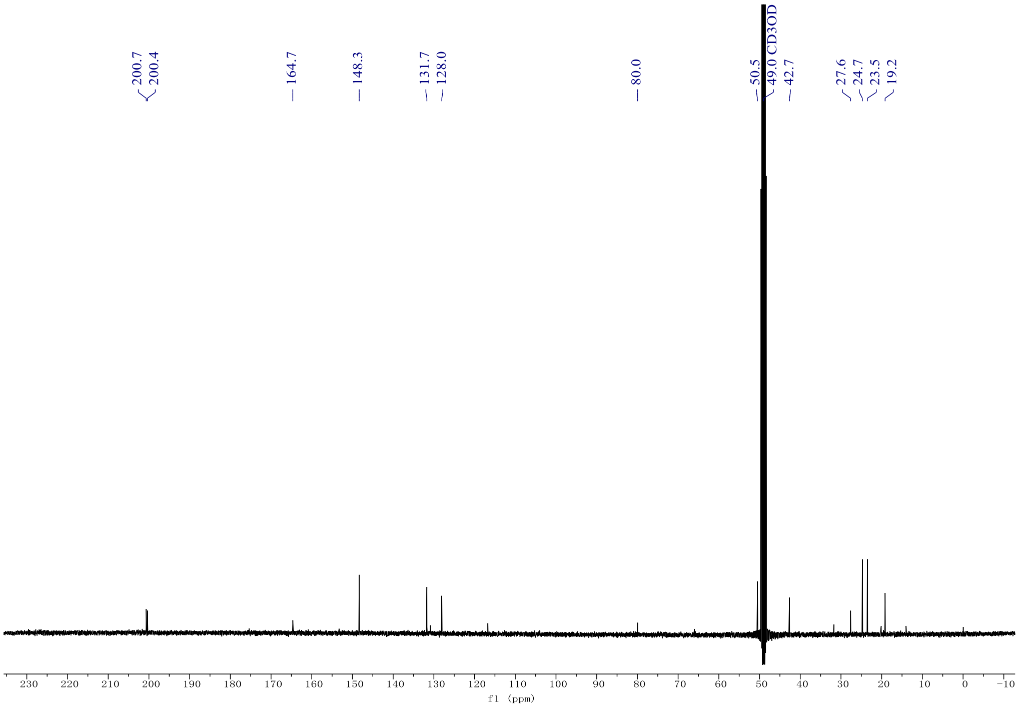

Figure S8-2. ^{13}C NMR spectrum (CD_3OD, 150 MHz) of compound **8**

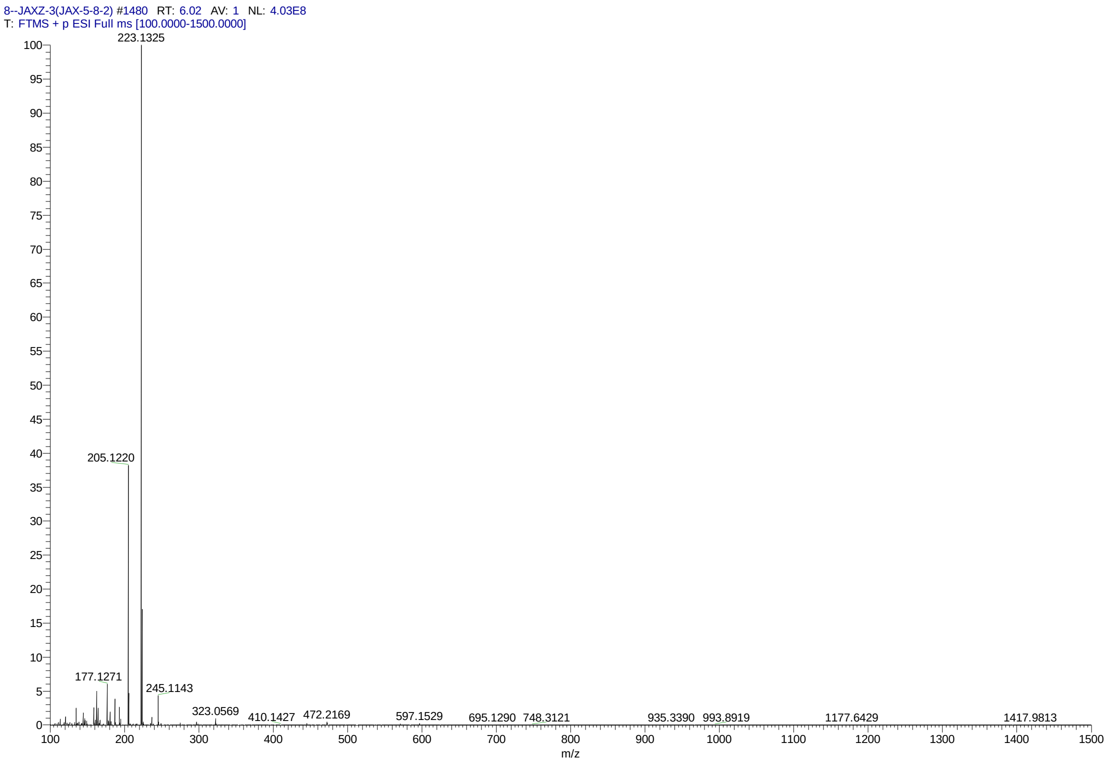

Figure S8-3. (+)-HRESIMS spectrum of compound **8**

Figure S9. NMR and MS of Compound 9

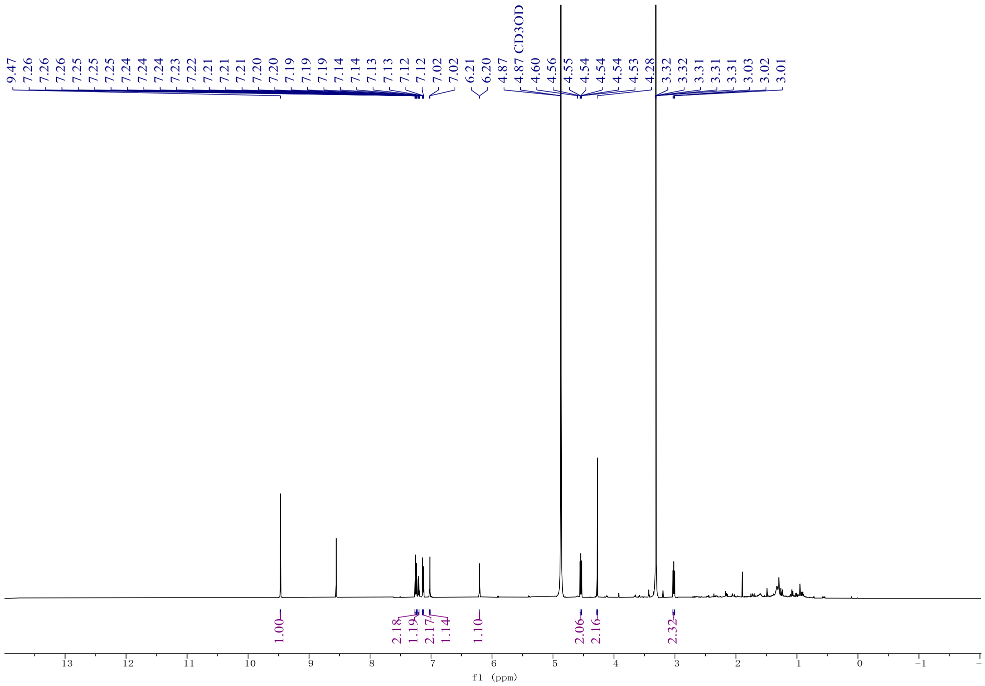

Figure S9-1. ^{1}H NMR spectrum (CD_3OD, 600 MHz) of compound **9**

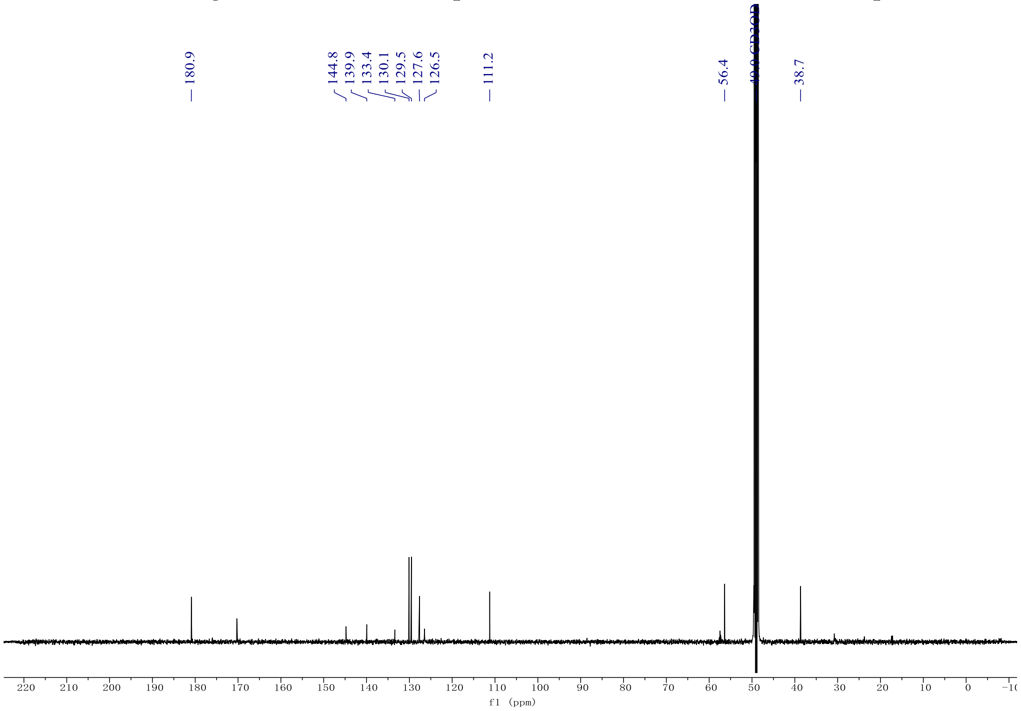

Figure S9-2. ^{13}C NMR spectrum (CD_3OD, 150 MHz) of compound **9**

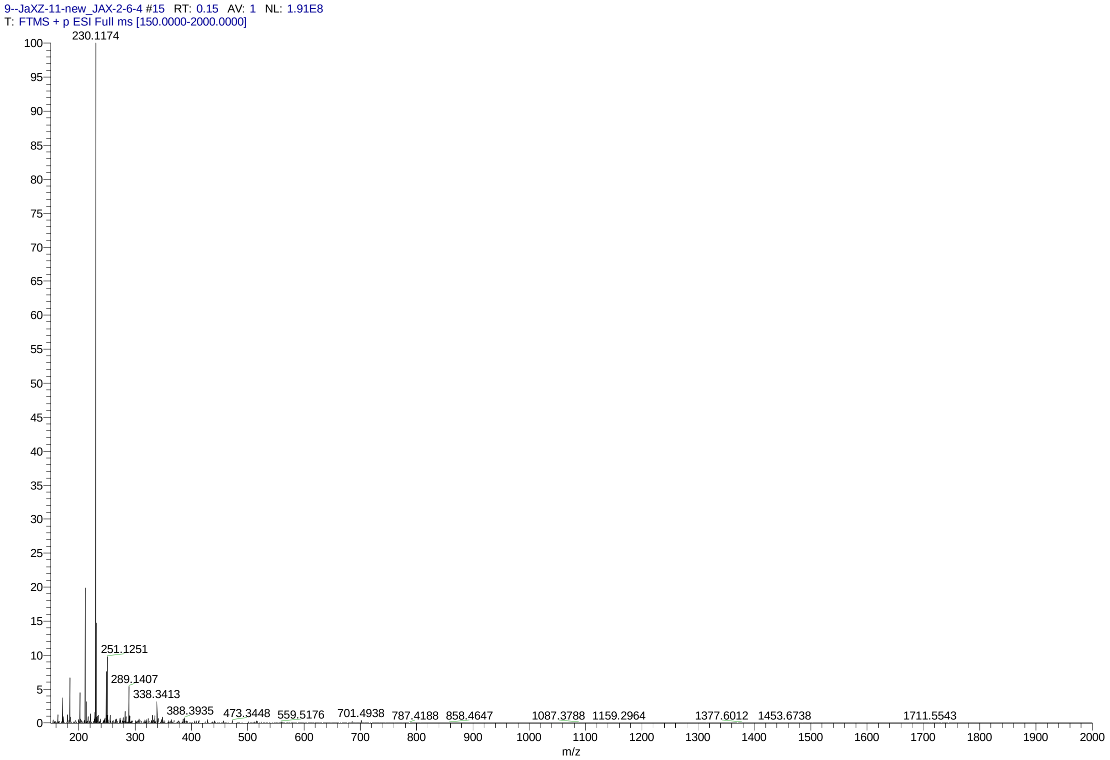

Figure S9-3. (+)-HRESIMS spectrum of compound **9**

Figure S10. NMR and MS of Compound 10

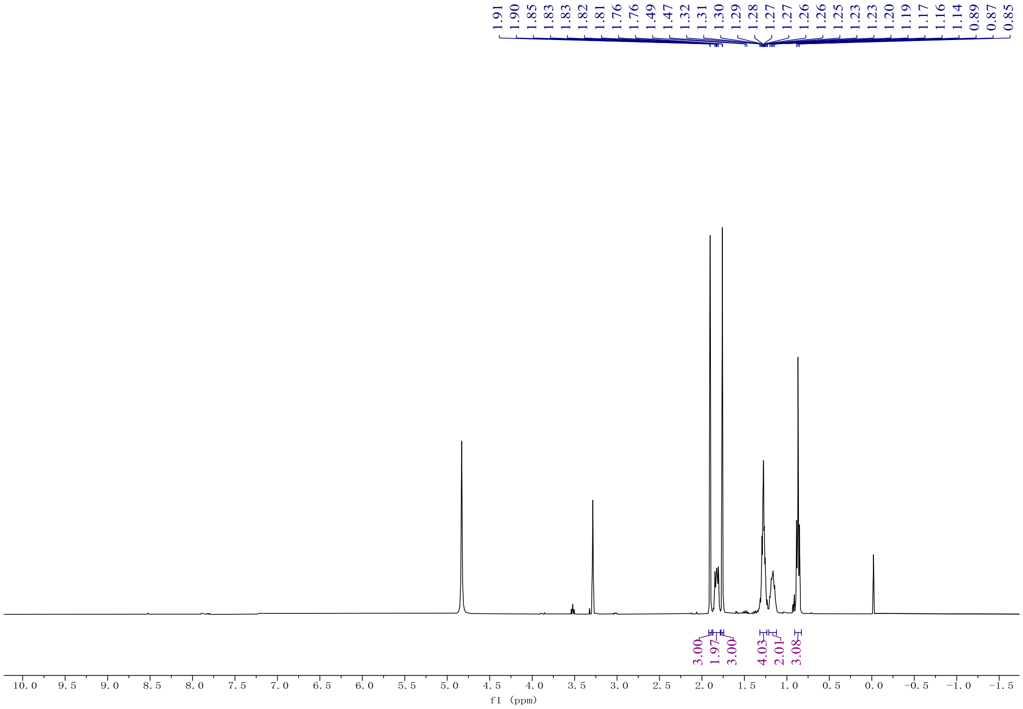

Figure S10-1. ^{1}H NMR spectrum (CD_3OD, 400 MHz) of compound **10**

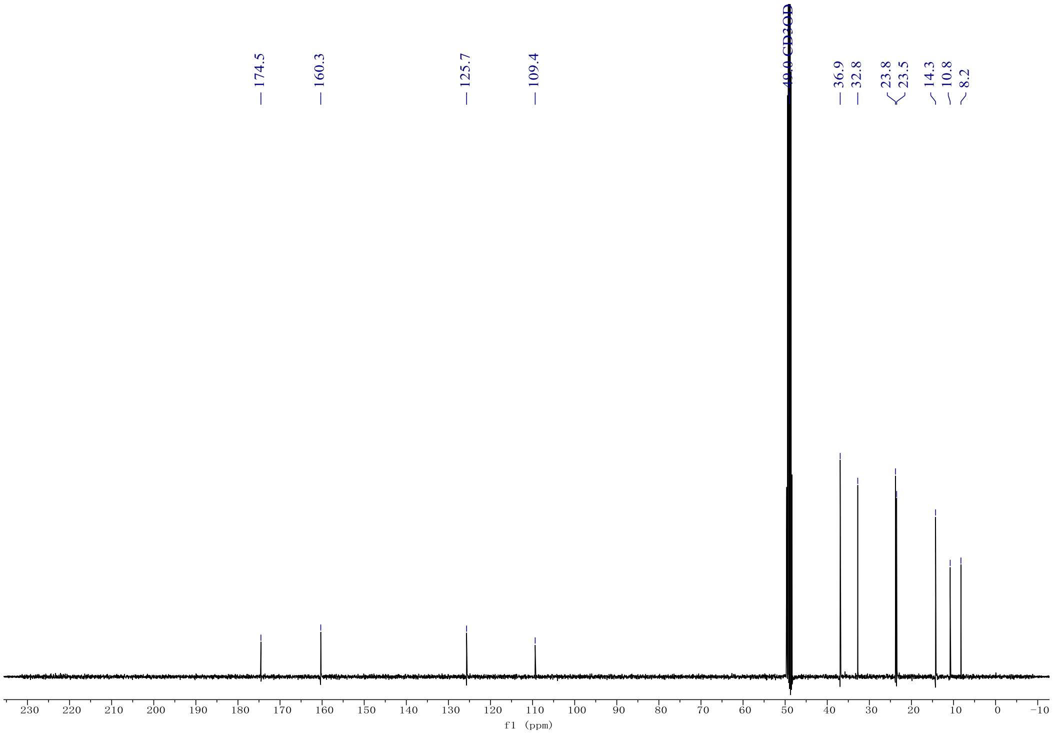

Figure S10-2. ^{13}C NMR spectrum (CD_3OD, 100 MHz) of compound **10**

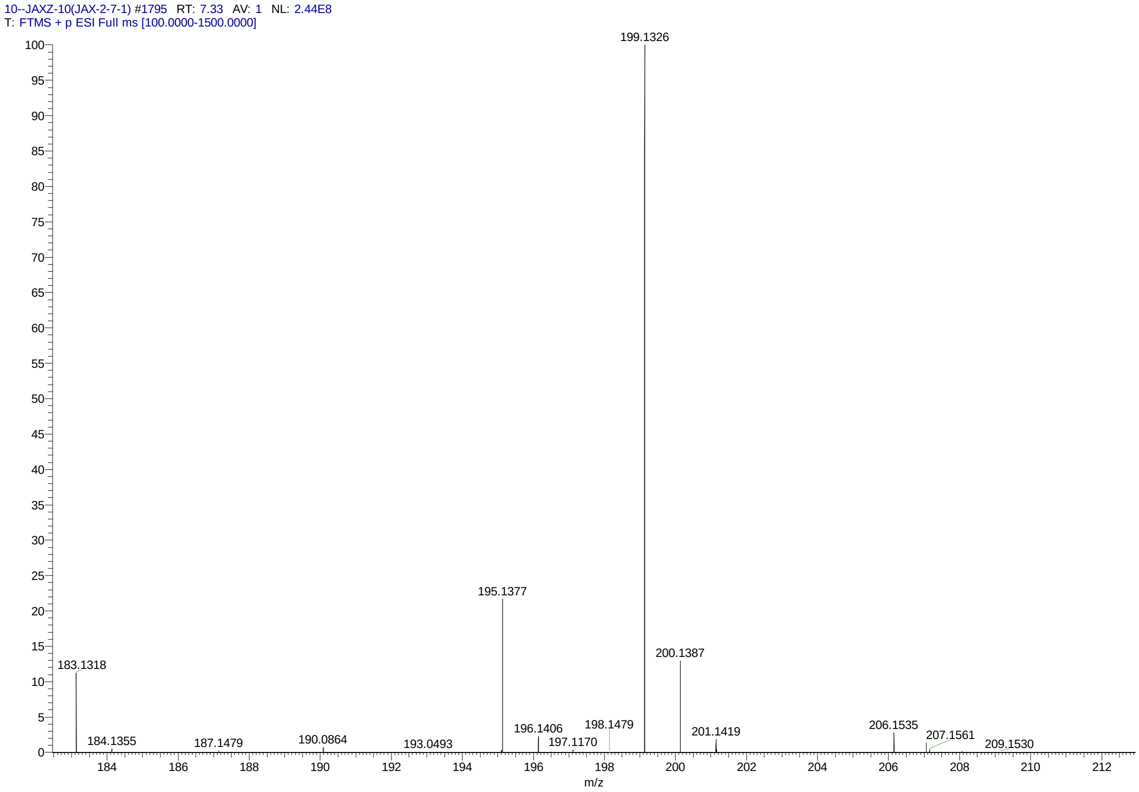

Figure S10-3. (+)-HRESIMS spectrum of compound **10**

Figure S11. NMR and MS of Compound 11

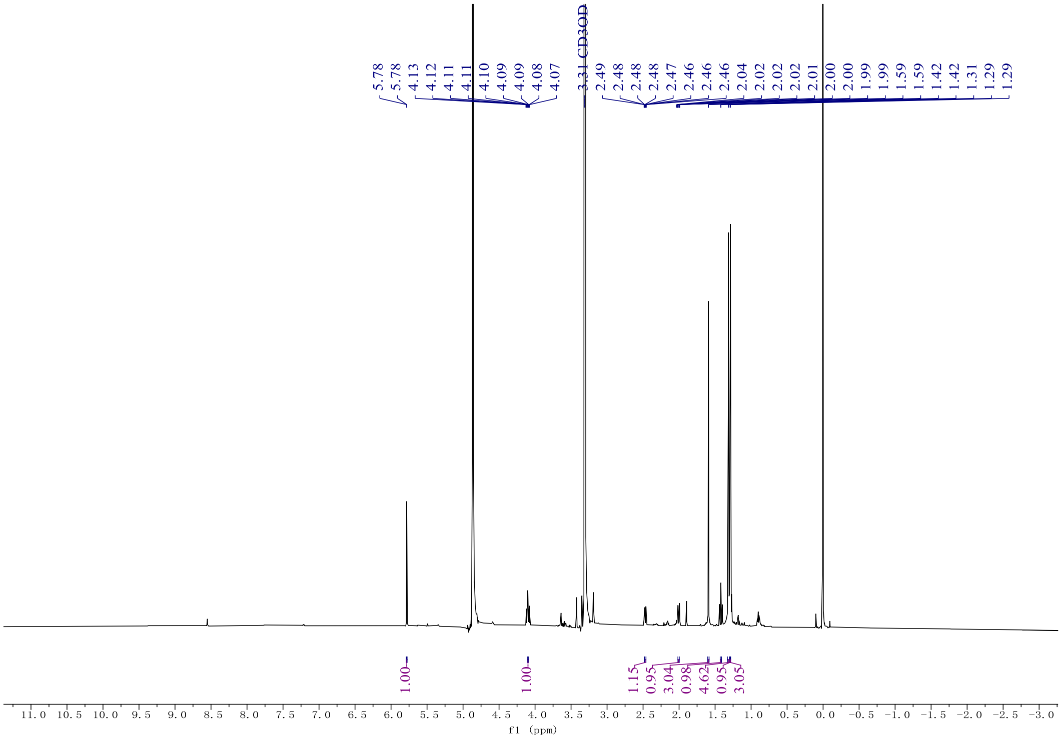

Figure S11-1. ^{1}H NMR spectrum (CD3OD, 600 MHz) of compound **11**

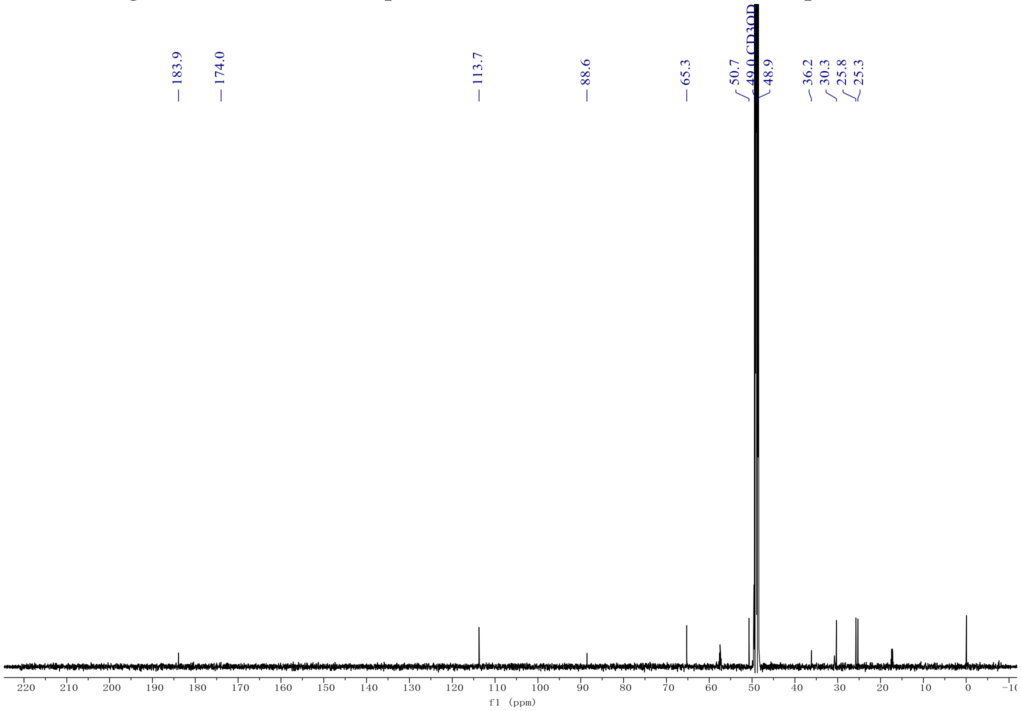

Figure S11-2. ^{13}C NMR spectrum (CD3OD, 150 MHz) of compound **11**

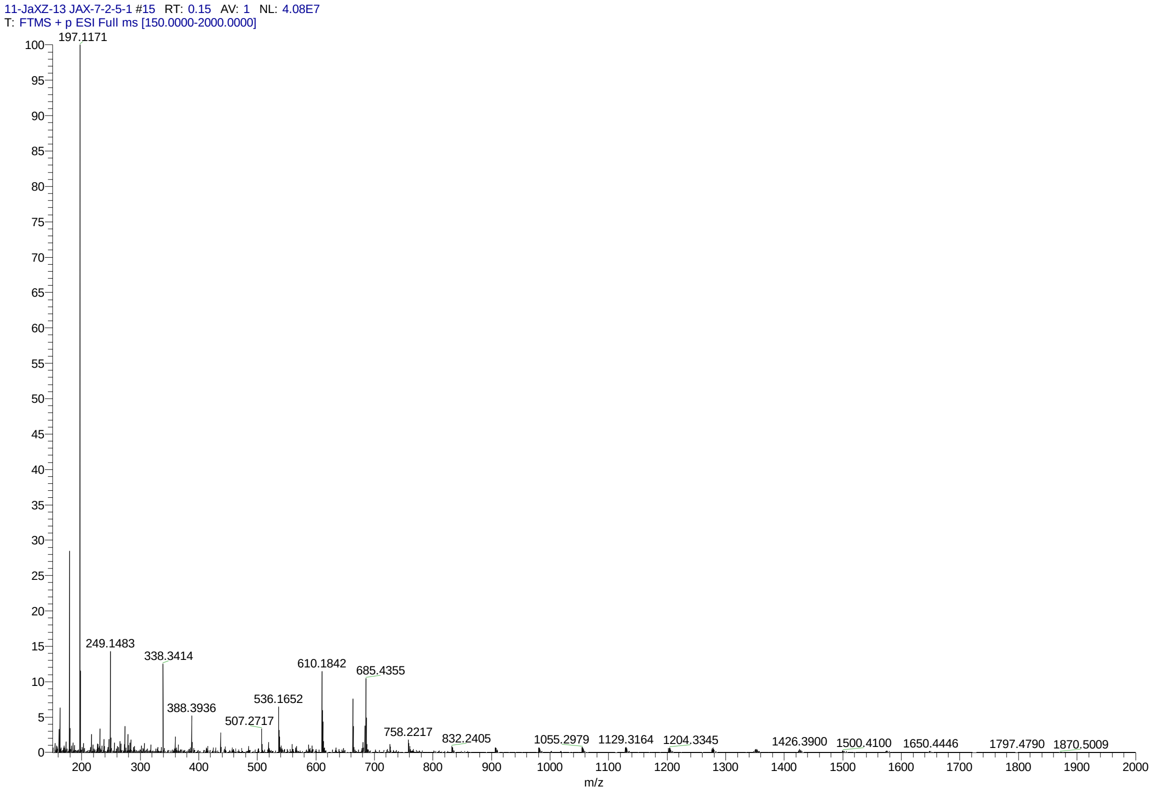

Figure S11-3. (+)-HRESIMS spectrum of compound **11**

Table S1. Equipment used for analyses

Experimental procedure	Equipment
NMR spectra	Bruker AVANCE III 600 spectrometer (Bruker, Bremerhaven, Germany) (600 MHz for ^{1}H and 150 MHz for ^{13}C NMR)<br>Varian VNMRS 600 spectrometer (Varian, CA, USA) (600 MHz for ^{1}H and 150 MHz for ^{13}C NMR)
HR-ESIMS	QSTAR Elite LC-MS/MS spectrometer (AB Sciex, MA, USA)
Optical rotations	Autopol VI automatic polarimeter (Rudolph Research Analytical, NJ, USA)
CD spectra	Chirascan spectropolarimeter (Applied Photophysics, UK)
UV spectra	Shimadzu UV-2550 spectrophotometer (Shimadzu, Japan)
IR spectra	Nicolet 6700 spectrometer (Thermo Fisher Scientific)
MPLC chromatography	Sepacore® flash systems X-50 (BUCHI, Switzerland)
Semi-preparative HPLC separations	Dionex UltiMate3000 pump system equipped with an UltiMate RS Variable Wavelength Detector (Dionex, Thermo Scientific, MA, USA)<br>XSelect CSH Prep C18 (5 μm OBD, 10 × 250 mm) column (Waters, MA, USA)
Silica gel, TLC plate	Yantai Jiangyou Silica Gel Development LTD, P. R. China, fractions were monitored by TLC chromatography, soaked in 10% ethanol sulfate chromogenic agent and heated appropriately for color development. All the solvents used were of analytical grade and obtained from Tansoole (Shanghai, China).
